# Tumor-derived exosomal *KPNA2* activates fibroblasts and interacts with KIFC1 to promote bladder cancer progression, a process inhibited by miR-26b-5p

**DOI:** 10.1186/s11658-025-00687-w

**Published:** 2025-02-16

**Authors:** Cong Yin, Cen Liufu, Shuai Ye, Tao Zhu, Jiahao Jiang, Mingxia Wang, Liqun Zhou, Lin Yao, Yan Wang, Bentao Shi

**Affiliations:** 1 Department of Urology, The First Affiliated Hospital of Shenzhen University, Shenzhen Second People’s Hospital, No. 3002, Sungangxi Road, Shenzhen, 518035 People’s Republic of China; 2 Present Address: Shantou University Medical College, Shantou, 515041 China; 3 Department of Urology, Peking University Shenzhen Hospital, Institute of Urology, Shenzhen PKU-HKUST Medical Center, Shenzhen, 518036 China; 4 Shenzhen University Health Science Center, Shenzhen, 518055 China; 5 Department of Urology, Shenzhen Second People’s Hospital, Clinical College of Anhui Medical University, Shenzhen, 518035 China; 6 The Fifth Clinical Medical College of Anhui Medical University, Hefei, 230032 Anhui China; 7 Department of Urology, Peking University First Hospital, No. 8 Xishiku St., Xicheng District, Beijing, 100034 People’s Republic of China; 8 Beijing Key Laboratory of Urogenital Diseases (Male) Molecular Diagnosis and Treatment Center, No. 8 Xishiku St., Xicheng District, Beijing, 100034 China

**Keywords:** BCa, Exosomes, *KPNA2*, Tumor microenvironment, Fibroblast activation, CAFs

## Abstract

**Background:**

Recent studies have illuminated the complexities of treating advanced bladder cancer (BCa), underscoring the importance of comprehending its molecular mechanisms for creating novel therapies. While the role of Karyopherin a2 (*KPNA2*) in promoting BCa growth is established, the precise mechanism remains elusive.

**Methods:**

To investigate the regulatory role of *KPNA2* in BCa, we employed a comprehensive approach integrating clinical case data and bioinformatics analysis to evaluate the expression of *KPNA2* in BCa tissues. Mechanisms promoting cancer by *KPNA2* were examined using both in vivo and in vitro models.

**Results:**

Our research reveals that miR-26b-5p acts as an anticancer factor by targeting and inhibiting *KPNA2* expression. Furthermore, we have observed that the interaction between *KPNA2* and Kinesin Family Member C1 (*KIFC1*) facilitates the transition of BCa cells into the G2/M phase, thereby promoting tumor advancement via activation of the Phosphoinositide 3-kinase (PI3K)- Protein Kinase B (AKT) pathway. Importantly, this investigation is the first to identify *KPNA2* expression in exosomes originating from BCa tissues. Plasma exosomes from patients with BCa exhibited notably increased levels of *KPNA2* compared with healthy controls, suggesting *KPNA2* as a potential new tumor indicator. Additionally, *KPNA2* from BCa cells triggered the conversion of fibroblasts into cancer-associated fibroblasts (CAFs), which secreted elevated levels of interleukin-6 (IL-6), contributing to a tumor-supporting environment.

**Conclusions:**

These findings suggest that *KPNA2* is a key gene that promotes BCa progression, can potentially be a novel tumor marker, and may serve as a new therapeutic target for BCa.

**Graphical Abstract:**

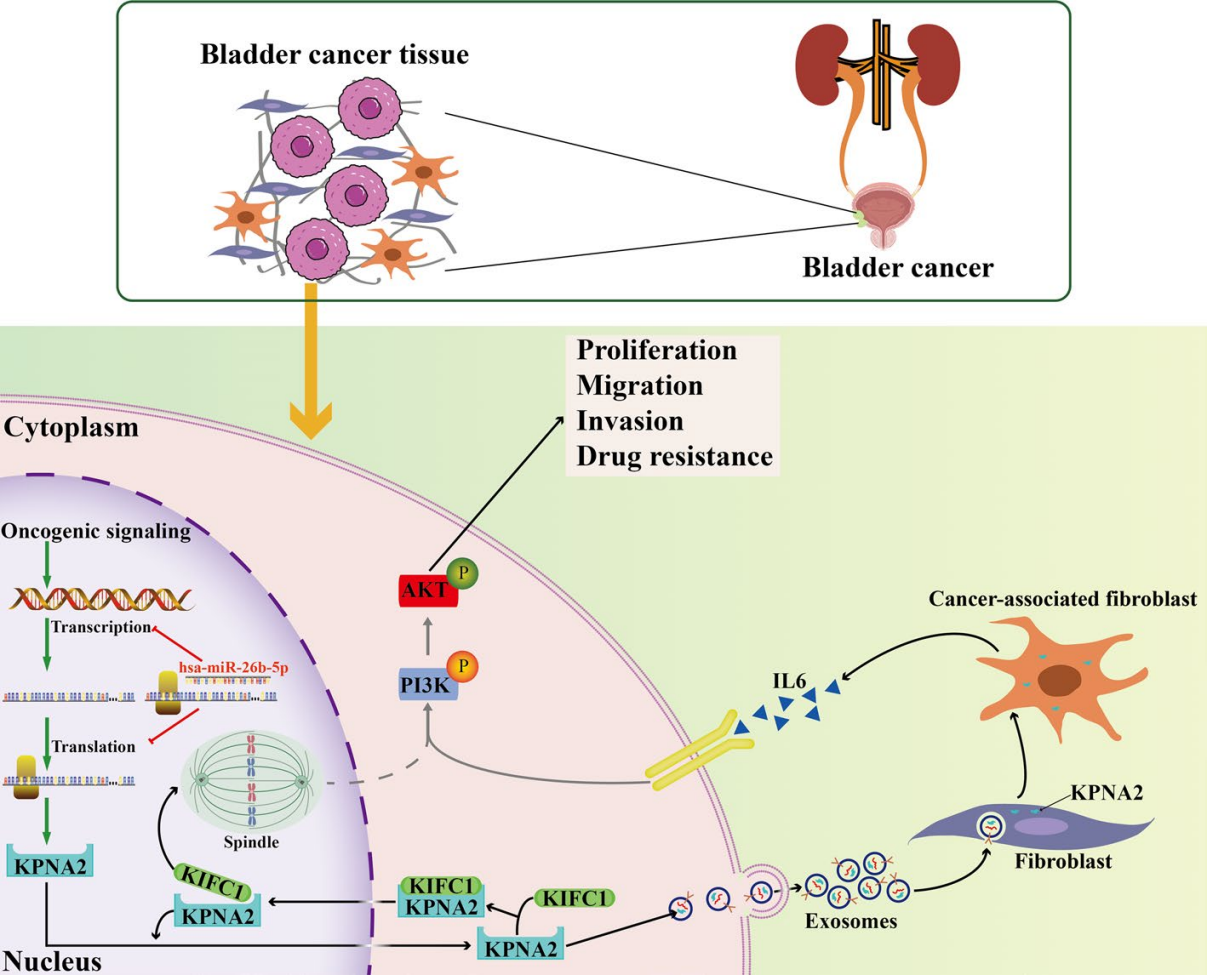

**Supplementary Information:**

The online version contains supplementary material available at 10.1186/s11658-025-00687-w.

## Introduction

Bladder cancer (BCa) is the ninth most common urological cancer globally, with approximately 60,000 new cases and 13,000 deaths annually in the USA. Over 90% of BCa cases originate from the transitional epithelium and often recur and progress [1]. BCa progression involves complex genetic and signaling interactions. Understanding these mechanisms is crucial for developing early detection and management strategies [2]. Exosomes, key mediators of cell communication, carry biomolecules, such as proteins, RNA, DNA, and lipids that influence recipient cells [3, 4]. In BCa, they modify the tumor microenvironment and regulate tumor dynamics by transporting and releasing tumor-promoting or inhibiting substances [5]. Thus, analyzing the molecular content and functions of BCa-related exosomes reveals tumor mechanisms and provides opportunities for early diagnosis and targeted therapy.

Our prior work links Karyopherin a2 (*KPNA2*) expression with increased BCa cell proliferation and migration, suggesting its role in BCa progression and metastasis [6–8]. Current findings further show that *KPNA2* expression correlates with CAF infiltration in tumors [9, 10]. CAFs, key players in the tumor microenvironment, enhance tumor growth by secreting cytokines and remodeling the extracellular matrix, indicating that *KPNA2* may drive tumor progression through CAF modulation [11, 12]. Elevated *KPNA2* levels in exosomes across various cancers, including lung and esophageal cancers, underscore its potential as a circulating marker [13, 14], but its precise role in BCa remains uncertain.

MicroRNAs (miRNAs) are conserved endogenous non-coding RNAs, approximately 22 nucleotides long [15], that regulate gene expression by binding to the 3′ untranslated region of messenger RNA (mRNAs), inhibiting translation or inducing mRNA degradation [16, 17]. Dysregulation of miRNA expression can lead to various diseases, particularly tumors. This study identifies miR-26b-5p as a tumor suppressor in BCa. We introduce KIFC1 as a partner of *KPNA2* and elucidate the miR-26b-5p/*KPNA2*/KIFC1 axis in BCa progression. Moreover, exosomal *KPNA2* in BCa tissues emerges as a promising diagnostic marker, aiding fibroblast transformation into CAFs and disrupting tumor interactions. These insights provide a basis for targeted diagnostic strategies.

## Material and method

### Patient samples

Samples were obtained from the Department of Urology at The First Affiliated Hospital of Shenzhen University, with the approval of the hospital’s ethics committee. Patients were fully informed about their sample nature, associated risks, study objectives, and provided written informed consent before participating in the experiments.

### Cell lines

Human epithelial cells SV-HUC-1 and BCa cells (TCCSUP, SW780, UMUC-3, T24, 5637, J82) were obtained from the American Type Culture Collection, while human fibroblast cells were from Saibaikon Biotech (Shanghai). SV-HUC-1 cells were cultured in Ham’s F-12 K medium, 5637 cells in 1640 medium, TCCSUP, UMUC-3, T24, SW780, and J82 cells in Dulbecco’s Modified Eagle’s Medium (Gibco, USA). Human fibroblast cells were grown in the PriMed-iCell-003 medium (Saibaikon). All cells were incubated at 37 °C in a humidified 5% CO_2_ atmosphere with 10% fetal bovine serum (FBS), 100 U/mL penicillin(Gibco), and 100 μg/mL streptomycin(Gibco).

### Antibody

p-PI3K (CST 17366), TSG101 (Abcam, ab125011), Alix (Abcam, ab186429), CD9 (Abcam, ab263019), Calnexin (Abcam, ab22595), cyclin B1 (CST 12231), β-tubulin (Abcam, ab6046), H3 (Abcam, ab1791), α-SMA (Proteintech, 67735-1-IG), PI3K (Santa, sc-1637), p-AKT (CST, 13038), AKT (CST, 11E7), *KPNA2* (Abcam, ab70160), and KIFC1 (Abcam, 172620), and Ki67 (CST, 9449).

### Database mining

The TCGA database (https://portal.gdc.cancer.gov/) and the GEO dataset GSE13507 (https://www.ncbi.nlm.nih.gov/) were utilized to obtain mRNA expression levels in BCa and their corresponding normal tissues. The GEO dataset GSE236933 (https://www.ncbi.nlm.nih.gov/) was utilized to obtain miRNA expression levels in BCa and their corresponding normal tissues.

### Screening for miRNAs targeting *KPNA2*

To identify differentially expressed miRNAs, we analyzed GEO’s miRNA expression data. We utilized online prediction tools, such as starBase (http://starbase.sysu.edu.cn/), miRTarBase (https://mirtarbase.cuhk.edu.cn/), and miRDB (https://mirdb.org/) to identify miRNAs targeting the *KPNA2* gene for potential regulation of its expression.

### In situ hybridization

Single-site tissue microarray (TMA) slides (HBlaU066Su01, Shanghai Outdo Biotech) were analyzed using the ISH detection kit mk11286-h and BOSTER probes (Wuhan). Staining intensity was categorized from 0 to 3 (negative to dark blue/purple) and confirmed by two pathologists.

### Immunohistochemistry (IHC)

IHC staining on single spot TMA slides (HBlaU066Su01, Shanghai Outdo Biotech) was performed using KIFC1 and *KPNA2* rabbit antibodies (1:1000, Abcam) with the rabbit streptavidin–biotin detection system (ZSGB-BIO). Staining was scored by proportion (1–4: 0–25%, 25–49%, 50–75%, and 75–100%, respectively) and intensity (0–3: negative, weak, moderate, and strong, respectively). Combined scores (0–12) categorized samples into high (7–12) and low (0–6) expression groups, confirmed by two pathologists.

### Immunofluorescence

Cells were inoculated on cell slides in 24-well plates. After 2 days, they were washed with phosphate buffered saline (PBS) and fixed with 4% paraformaldehyde for 30 min. After rinsing with PBS and permeabilizing/blocking with 5% bovine serum albumin (BSA) and 0.5% Triton X-100 for 1 h, cells were incubated with primary antibody in 5% BSA at 4 °C overnight. Following PBS rinses, cells were incubated with a fluorescent secondary antibody (Servicebio) in 5% BSA for 1 h in darkness, stained with DAPI for 10 min, washed with PBS, and observed via fluorescence microscopy.

### RT-qPCR

RNA was isolated from cultured cells and tissues using Trizol Reagent (Takara). Total RNA was quantified with a SYBR Premix Ex Taq™ II kit (Takara) on a LightCycler 480 (Roche) following the manufacturer’s protocol. Cycling threshold (CT) data were collected and averaged, with comparisons made to controls using the comparative CT method. U6 snRNA and human GAPDH were internal controls for miRNA and mRNA, respectively. Normalized quantities were calculated using the 2-ΔΔCT method and analyzed with GraphPad Prism 6. The primer sequences, synthesized by Sangon Biotech (Shanghai), are listed in Additional file 2: Table S1.

### Western blot

Proteins were lysed in ice-cold radioimmunoprecipitation assay (RIPA) buffer, separated by 10% sodium dodecyl sulfate polyacrylamide gel electrophoresis (SDS-PAGE), and transferred to polyvinylidene fluoride (PVDF) membranes (Millipore). The membranes were blocked with 5% skimmed milk for 2 h at room temperature, washed three times with tris-buffered saline with Tween 20 (TBST; 10 min each), and incubated overnight at 4 °C with the corresponding primary antibodies. GAPDH served as the control. After TBST washes, membranes were incubated with horseradish peroxidase (HRP)-labeled anti-rabbit or anti-mouse immunoglobulin G (IgG) for 1 h at room temperature. Signals were detected using an enhanced chemiluminescent substrate (Millipore) and a chemiluminescent imaging system (Tanon-5200Multi, Tanon).

### Transient transfections

Cells in six-well plates were cultured to 30–50% confluence for transfection. Lipofectamine 3000 (Invitrogen, USA) was combined with the respective plasmids, mimics, inhibitors, or small interfering RNAs (siRNAs; GenePharma, China) in opti-MEM for 15 min, then added to each well. Transfection was performed for 24 h, followed by a 48-h recovery period, after which RNA, proteins, and cells were collected for analysis. The sequences of each target are listed in Additional file 3: Table S2.

### Dual luciferase reporter assay

Next, we retrieved the complete *KPNA2* sequence from the gene library and design primers to amplify the *KPNA2* 3′ untranslated region (UTR). Conducted XhoI and NotI double digestion of the psiCHECK2 vector, then ligated the *KPNA2* 3′ UTR to create the psiCHECK2-*KPNA2* 3′ UTR vector (Gene Pharma, China). The vector was transformed into competent *Escherichia coli* and the psiCHECK2-*KPNA2* 3′ UTR dual luciferase reporter plasmid was amplified. Lipofectamine 3000 (Invitrogen, USA) was used to transfect miRNA mimics and inhibitors (Gene Pharma, China) into cells for 48 h. Luciferase activity was assessed using the dual-luciferase reporter assay system (Promega, USA), calculating relative activity (Renilla/Firefly) with firefly luciferase as the reference for reporter gene expression.

### Cell counting kit-8 (CCK-8) assay

Cells were seeded at 1500 cells per well in 96-well plates. Ten microliters of CCK-8 reagent were added at 0, 24, 48, and 72 h post-seeding, in darkness, according to the manufacturer’s instructions. After 3 h of incubation, optical density (OD) at 450 nm was measured using a microplate reader.

### Colony formation assay

Cells were seeded in six-well plates at a density of 1500 cells per well and cultured for 10 days. After fixation with 4% paraformaldehyde and staining with 0.1% crystal violet for 30 min, the colonies were washed with PBS and imaged. The crystal violet stain was then eluted with 33.3% glacial acetic acid, and the absorbance was measured at 590 nm using a microplate reader.

### Cell migration and invasion assay

In the wound healing assay, cells in six-well plates formed monolayers, after which wounds were created using a 200 µL pipette, and migration was assessed at 0 and 24 h. Transwell chambers (CoStar, USA) were utilized for the transwell assays following the manufacturer’s instructions. Cells were fixed and stained with 0.2% crystal violet to visualize migration and invasion. Absorbance was measured after elution of the dye with 33.3% glacial acetic acid and transferred to a 96-well plate.

### Cell apoptosis assay

Cells were centrifuged, washed twice with precooled PBS at 4 °C, and resuspended in 500 μL of binding buffer to achieve a concentration of 1 × 10^7^ cells/mL. Subsequently, 100 μL of this suspension was mixed with 5 μL of Annexin V-FITC (Dojindo) and 5 μL of Propidium iodide (PI), followed by a 15-min incubation at room temperature in the dark. Apoptosis data were analyzed via flow cytometry using FlowJo V10 software. Experiments were conducted in triplicate.

### Cell cycle analysis

Cells were harvested and washed with ice-cold PBS twice. Subsequently, the cells were fixed by incubating in 1 ml of precooled 75% ethanol at 4 °C overnight. The target cells were stained with PI/RNase staining buffer (BD Biosciences) for 30 min in the dark at room temperature. The DNA content was assessed using flow cytometry, and the data was analyzed with FlowJo V 10 software. All experiments were conducted a minimum of three times.

### Chemosensitivity assay

Cells were seeded into 96-well plates at 3000 cells per well to assess sensitivity to doxorubicin (Sigma). After an overnight incubation, the cells were exposed to different concentrations of doxorubicin in a culture medium. Cell viability was measured after 48 h using the CCK-8 assay. The experiment was independently repeated at least three times for reliability. Maintaining aseptic techniques and safe drug handling was essential.

### HDOCK prediction

We obtained the PDB structures of the corresponding proteins from the PDB database, predicted the direct interactions between the two proteins using HDOCK protein–protein, and analyzed the free energy between the two proteins using PRODIGY. The predicted multimeric structures obtained were analyzed using pymol v2.5.0 to obtain amino acid pairs that can interact to form hydrogen bonds between proteins and proteins.

### Coimmunoprecipitation

Coimmunoprecipitation was performed using an Invitrogen kit (USA) with proteins extracted in NP-40 lysis buffer (Beyotime, China). Cell lysates were incubated overnight at 4 °C with protein A/G agarose beads and either anti-*KPNA2* or anti-KIFC1 antibodies. Immunoprecipitation was facilitated by magnetic beads, followed by washing with pre-chilled RIPA lysis buffer. Bound proteins were then boiled and subjected to SDS-PAGE. Western blotting followed, using anti-*KPNA2* or anti-KIFC1 antibodies.

### Tissue exosome isolation and protein profiling

To promote dissociation, 200 mg of tissue was sliced on dry ice and incubated at 37 °C for 10–15 min. Then, the tissue was filtered using a 70 μm mesh to remove debris and centrifuge at 300 × *g* for 10 min and at 10,000 × *g* for 20 min, both at 4 °C. The supernatant was filtered through a 0.22 μm filter. Ultracentrifugation was performed at 150,000 × *g* for 2 h at 4 °C. The pellet was resuspended in 1 mL PBS and purified using the Exospur® kit (Echobiotech, China). The exosomes were concentrated to 200 μL with a 100 kDa MWCO Amicon® Ultra filter (Merck, Germany). Exosomes were identified by transmission electron microscopy and analyze protein markers via western blot. Size distribution and concentration were measured using a Nano Flow Cytometer (Flow NanoAnalyzer, NanoFCM Inc, China). Purify and extract proteins from exosomes, quantify them, perform SDS-PAGE, and conduct enzymatic digestion. Analyze the samples with mass spectrometry as detailed in Additional file 4: Table S3.

### Exosome tracing

Exosomes were purified and labeled with the PKH67 green fluorescent kit (Sigma-Mini67, Germany). The washed exosomes were suspended in Diluent C and gently mixed with PKH67, followed by the addition of an equal volume of FBS to bind excess dye. The labeled exosomes were incubated with target cells for 24 h. After nuclear staining, the uptake of exosomes by target cells was monitored with a confocal microscope (Sunyu, China).

### In vivo xenograft model

The animal experimental protocol received approval from the animal ethics committee of the First Affiliated Hospital of Shenzhen University. We utilized two cohorts of 4–6 week-old Balb/c nude mice to assess the in vivo effects of the target genes. In the first cohort of 20 mice, we employed a random number generator to assign them to four groups: a lentiviral control group, a miR-26b-5p overexpression group, a *KPNA2* overexpression group, and a miR-26b-5p plus *KPNA2* group. Each mouse received a subcutaneous injection of 3.5 × 10^6^ cells. Tumor diameters were measured with digital calipers, and mice were euthanized after 25 days for tumor photography and pathological examination. In the second cohort, mice were divided into two groups and injected with bladder cells either overexpressing or not expressing KIFC1. Tumor sizes were measured similarly, and mice were euthanized after 22 days.

### Statistical analysis

The statistician performed data analyses independently. Correlations between KIFC1 and *KPNA2* expression and clinical pathologic variables were analyzed using the χ2 test and Fisher’s exact test. For normally distributed samples, a two-tailed *t*-test was used to compare group means, while the Mann–Whitney *U* test was applied for non-normally distributed samples. One-way ANOVA, followed by Tukey’s test, was utilized to compare means among three or more groups, with *p* < 0.05 indicating statistical significance. All analyses were conducted using SPSS 20.0 and GraphPad Prism 6.

## Results

### MiR-26b-5p is lowly expressed in BCa and targets *KPNA2* regulation

**Fig. 1:**
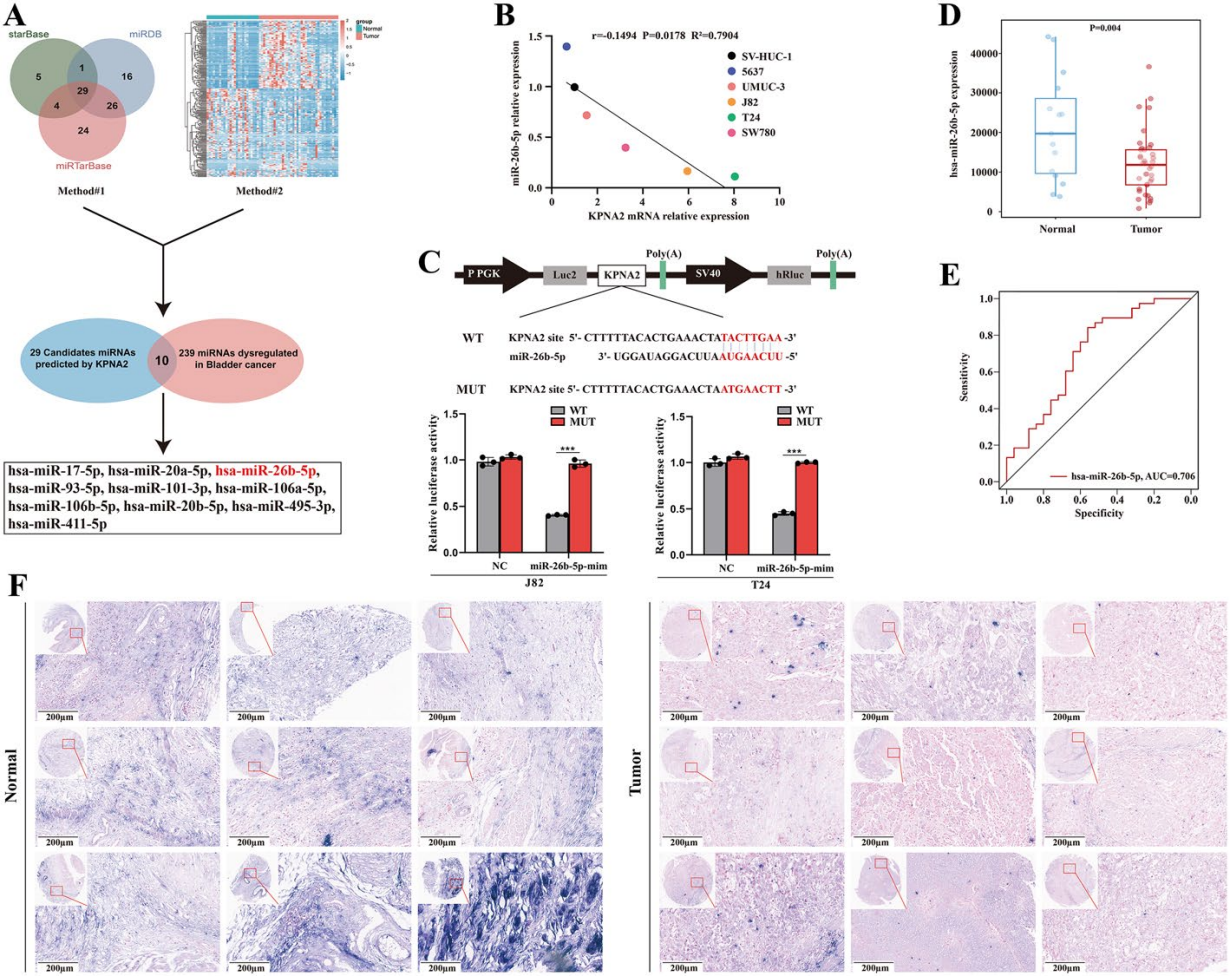
Systematic screening and validation of miRNAs targeting the *KPNA2* gene. **A** Online prediction software, including starBase, miRTarBase, miRDB, and miRNA Expression Database (GEO accession no. GSE236933, red denotes up-regulation, blue denotes down-regulation), identified 10 miRNAs likely targeting *KPNA2* and showing low expression in BCa. **B** Quantitative reverse transcription polymerase chain reaction (RT-qPCR) analysis of miR-26b-5p expression in BCa cell lines (5637, UMUC-3, J82, T24) and normal bladder epithelial cells (SV-HUC-1) demonstrated its regulatory relationship with *KPNA2*. **C** The dual luciferase reporter assay confirmed the direct binding relationship between miR-26b-5p and *KPNA2*. **D** GEO database microarray data revealed significant downregulation of miR-26b-5p in BCa tissues compared with normal bladder tissues (*p* = 0.004). **E** The ROC curve (AUC = 0.706) indicated good specificity in the GEO database. **F** Tissue microarrays, validated with clinical samples, showed significantly lower miR-26b-5p expression in BCa tissues compared with normal tissues (high expression in dark purple or dark blue, low expression in pink; *p* < 0.001)

To explore the miRNA regulatory mechanisms of *KPNA2* in BCa, we analyzed the miRNA microarray dataset GSE236933. Utilizing miRNA target prediction tools, such as starBase, miRTarbase, and miRDB, we identified 239 miRNAs with reduced expression in BCa tissues. Further intersection analysis narrowed down 29 candidates potentially involved in regulating *KPNA2*. Through a comprehensive bioinformatics approach, we selected ten miRNAs for validation: hsa-miR-17-5p, hsa-miR-20a-5p, hsa-miR-26b-5p, hsa-miR-93-5p, hsa-miR-101-3p, hsa-miR-106a-5p, hsa-miR-106b-5p, hsa-miR-20b-5p, hsa-miR-495-3p, and hsa-miR-411-5p (Fig. 1A).

RT-qPCR analysis of uroepithelial and BCa cell lines revealed a significant negative correlation between miR-26b-5p and *KPNA2* expression (Fig. 1B, Additional file 1: Fig. S1). The regulatory role of miR-26b-5p on *KPNA2* was confirmed using a dual-luciferase reporter assay (Fig. 1C). Analysis of the GSE236933 dataset indicated that miR-26b-5p expression was significantly lower in BCa tissues compared with normal tissues, and ROC curves validated the reliability of the data (Fig. 1D-E). Tissue microarrays further corroborated reduced miR-26b-5p levels in BCa tissues (Fig. 1F). These findings suggest that miR-26b-5p may regulate *KPNA2* in BCa, warranting further investigation.

### MiR-26b-5p inhibits the proliferative ability of BCa cells by suppressing *KPNA2* expression

**Fig. 2:**
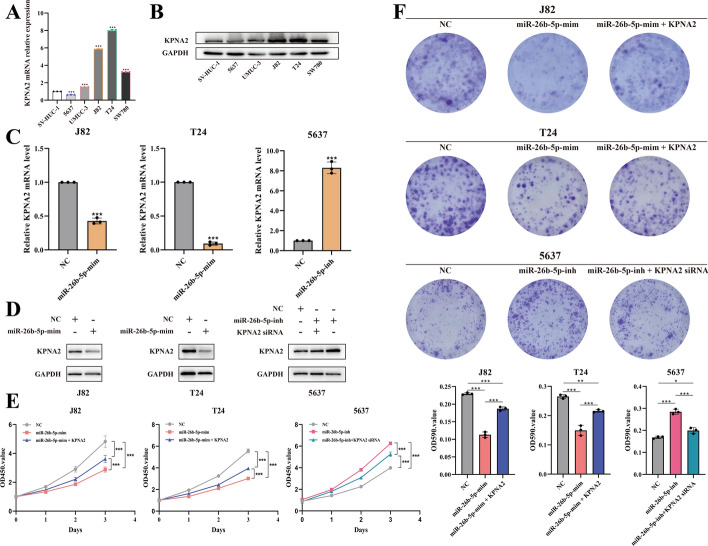
MiR-26b-5p inhibits the growth and proliferation ability of BCa cells. **A** *KPNA2* levels were analyzed using RT-qPCR in five BCa cell lines (5637, UMUC-3, J82, T24, and SW780) and an immortalized normal uroepithelial cell line (SV-HUC-1). **B** Western blot analysis of *KPNA2* protein abundance in the same five BCa cell lines and the immortalized normal urinary tract epithelial cell line (SV-HUC-1). **C** Validation of the transfection efficiency of miR-26b-5p mimic in J82 and T24 cell lines and miR-26b-5p inhibitor in 5637 cell lines using RT-qPCR. **D** *KPNA2* protein abundance was verified by western blot in J82 and T24 cell lines with miR-26b-5p-specific mimics transfected and 5637 cell lines with miR-26b-5p-specific inhibitor transfected. **E**, **F** CCK-8 assays and colony formation assays showed that miR-26b-5p mimic inhibited the growth and proliferation ability of J82 and T24 cells, and miR-26b-5p inhibitor promoted the growth and proliferation ability of 5637 cells. *, *p* < 0.05; **, *p* < 0.01; ***, *p* < 0.001

To investigate miR-26b-5p’s biological function in BCa cell lines, we measured its expression using qPCR across five BCa cell lines. miR-26b-5p was low expression in T24 and J82 lines and high in 5637, compared with SV-HUC-1 (Fig. 1B, Fig. 2A). On the basis of *KPNA2* expression levels (Fig. 2B), T24, J82 (low miR-26b-5p), and 5637 (high miR-26b-5p) cell lines were selected for further studies. Primers for specific miR-26b-5p mimics and inhibitors were designed (Additional file 3: Table S2). Overexpression of miR-26b-5p significantly decreased *KPNA2* protein levels, while inhibition increased them (Fig. 2C-D).

Functional assays showed that miR-26b-5p overexpression inhibited the proliferation of T24 and J82 cells, while the supplementation of *KPNA2* could partially reverse this effect. In contrast, silencing miR-26b-5p in 5637 cells promoted cell proliferation, and simultaneously knocking out *KPNA2* somewhat reduced this effect (Fig. 2E-F). These results indicate a negative regulatory relationship between miR-26b-5p and *KPNA2*.

### MiR-26b-5p inhibits migratory motility and enhances chemosensitivity in BCa cells by suppressing *KPNA2* expression

**Fig. 3:**
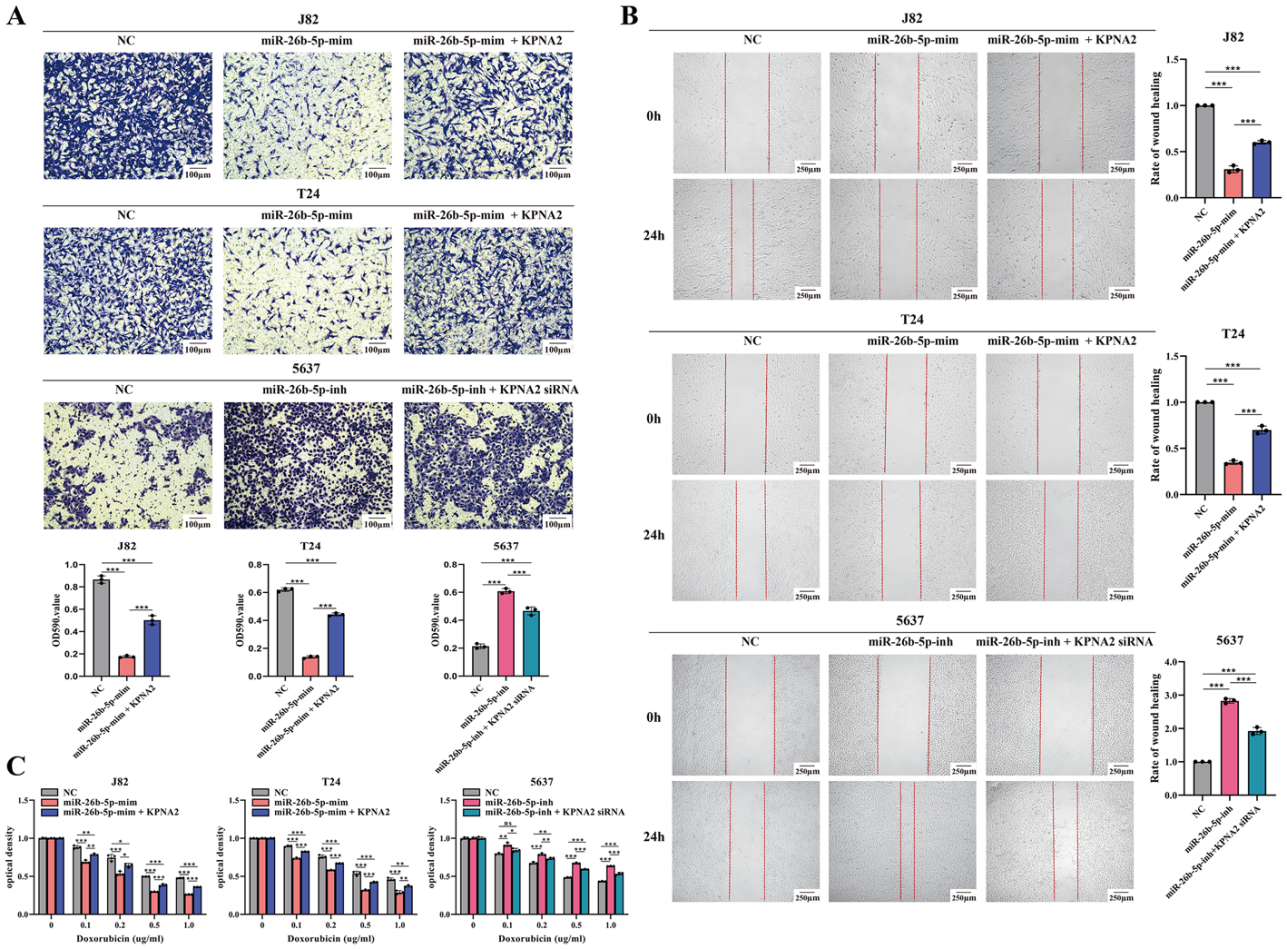
MiR-26b-5p inhibits migratory motility and enhances chemosensitivity of doxorubicin in BCa cells. **A**, **B** Wound healing assay and transwell invasion assay showed that miR-26b-5p mimic inhibited the migration and invasion ability of J82 and T24 cells, while the miR-26b-5 inhibitor enhanced the migration and invasion ability of 5637 cells. **C** The miR-26b-5p mimic significantly increased the chemosensitivity of J82 and T24 cells to doxorubicin, and the miR-26b-5p inhibitor decreased the chemosensitivity of 5637 cells to doxorubicin. *, *p* < 0.05; **, *p* < 0.01; ***, *p* < 0.001

Wound healing and transwell invasion assays indicated that miR-26b-5p overexpression significantly reduced the migration and invasion abilities of T24 and J82 cells, underscoring its role in inhibiting cell motility. *KPNA2* supplementation partially reversed these effects, confirming their negative regulatory relationship. In contrast, inhibiting miR-26b-5p enhances the migration and invasion capabilities of 5637 cells, underscoring its regulatory significance. Simultaneously depleting *KPNA2* also partially reversed the increased migration and invasion caused by the inhibition of miR-26b-5p, further confirming their interaction (Fig. 3A, B).

Additionally, miR-26b-5p enhanced BCa cell sensitivity to doxorubicin, while *KPNA2* re-expression induced resistance (Fig. 3C). These results suggest that miR-26b-5p functions as a tumor suppressor in BCa by suppressing *KPNA2* expression.

### MiR-26b-5p slows down cell division and promotes apoptosis by suppressing *KPNA2* expression

**Fig. 4:**
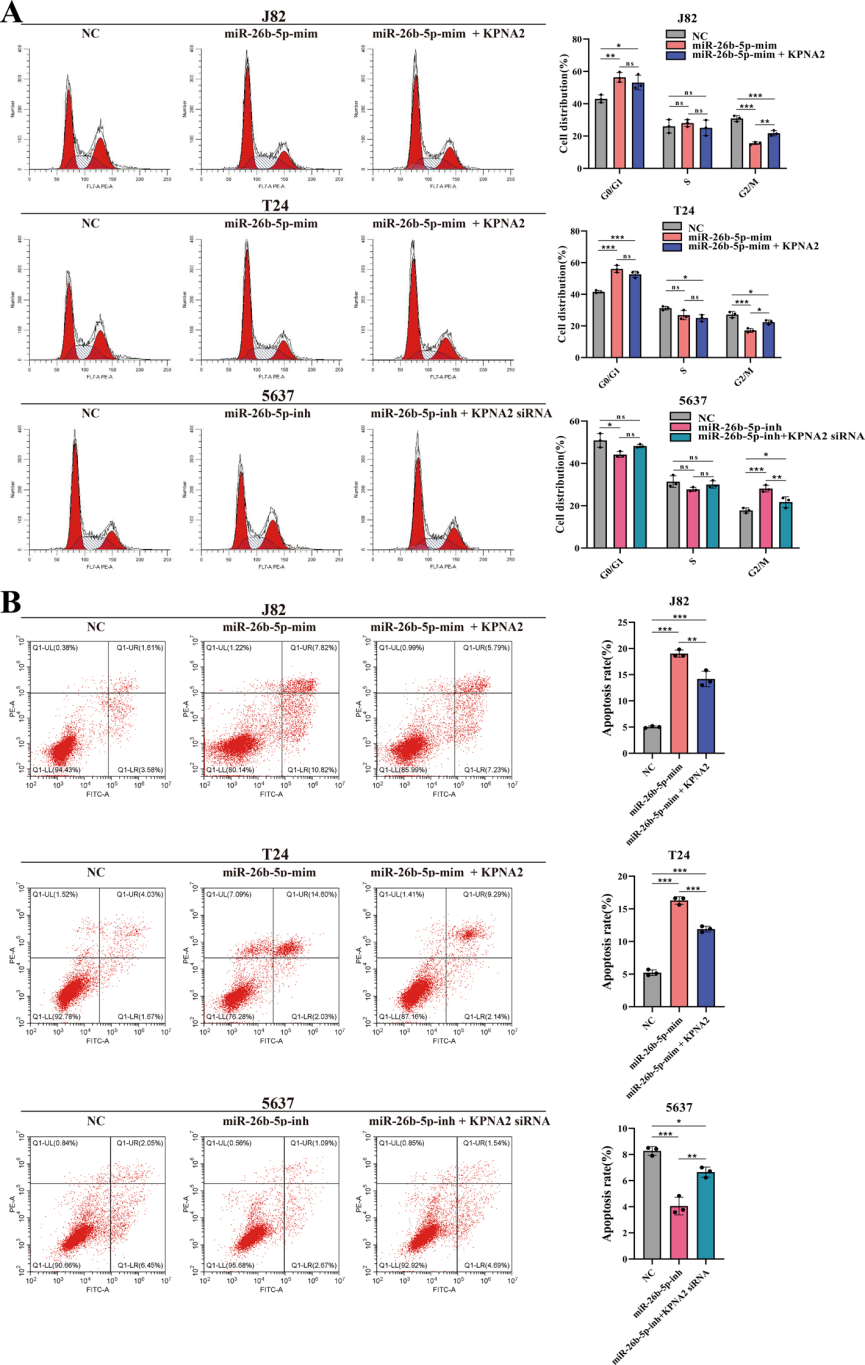
MiR-26b-5p induces G2/M phase arrest and promotes BCa cell apoptosis. **A** Using flow cytometry to investigate the impact of miR-26b-5p and *KPNA2* on the cell cycle of BCa cells. The study results indicated that overexpression of miR-26b-5p in J82 and T24 cells significantly reduced the number of cells in the G2/M phase, while supplementation of *KPNA2* partially reversed this effect. Conversely, inhibiting miR-26b-5p in 5637 cells led to a significant increase in cells in the G2/M phase, which could be partially counteracted by targeted knockout of *KPNA2*. **B** In apoptosis assays, higher levels of miR-26b-5p led to a notable rise in the proportion of apoptotic cells in J82 and T24 cells. Introducing exogenous *KPNA2* partially reverses this impact, leading to a reduction in the proportion of apoptotic cells. Lowering miR-26b-5p expression reduces the proportion of apoptotic cells in 5637 cells, whereas the depletion of *KPNA2* partially reinstates the proportion of apoptotic cells. *, *p* < 0.05; **, *p* < 0.01; ***, *p* < 0.001

MiR-26b-5p inhibits cell division and enhances apoptosis by repressing *KPNA2* expression. Flow cytometry analysis showed that miR-26b-5p upregulation significantly shortened the G2/M phase in J82 and T24 BCa cells, indicating its role in impeding cell cycle progression and slowing cell division. Co-overexpression of *KPNA2* with miR-26b-5p mimics partially reversed this effect. Conversely, inhibiting miR-26b-5p in 5637 cells extended the G2/M phase, promoting cell division and growth, an effect partially reversed by simultaneous *KPNA2* knockout (Fig. 4A). Apoptosis experiments indicate that increasing miR-26b-5p levels raises the percentage of apoptotic cells in J82 and T24 cells. At the same time, co-expression with *KPNA2* somewhat reduces apoptosis, suggesting that *KPNA2* counteracts the pro-apoptotic effect of miR-26b-5p. In 5637 cells, reducing miR-26b-5p decreased apoptosis, but the knockout of *KPNA2* partially restored apoptosis levels. This further emphasizes the role of miR-26b-5p in inhibiting bladder cancer progression by suppressing *KPNA2* levels (Fig. 4B).

### *KPNA2* imports KIFC1 into the nucleus of BCa cells

**Fig. 5:**
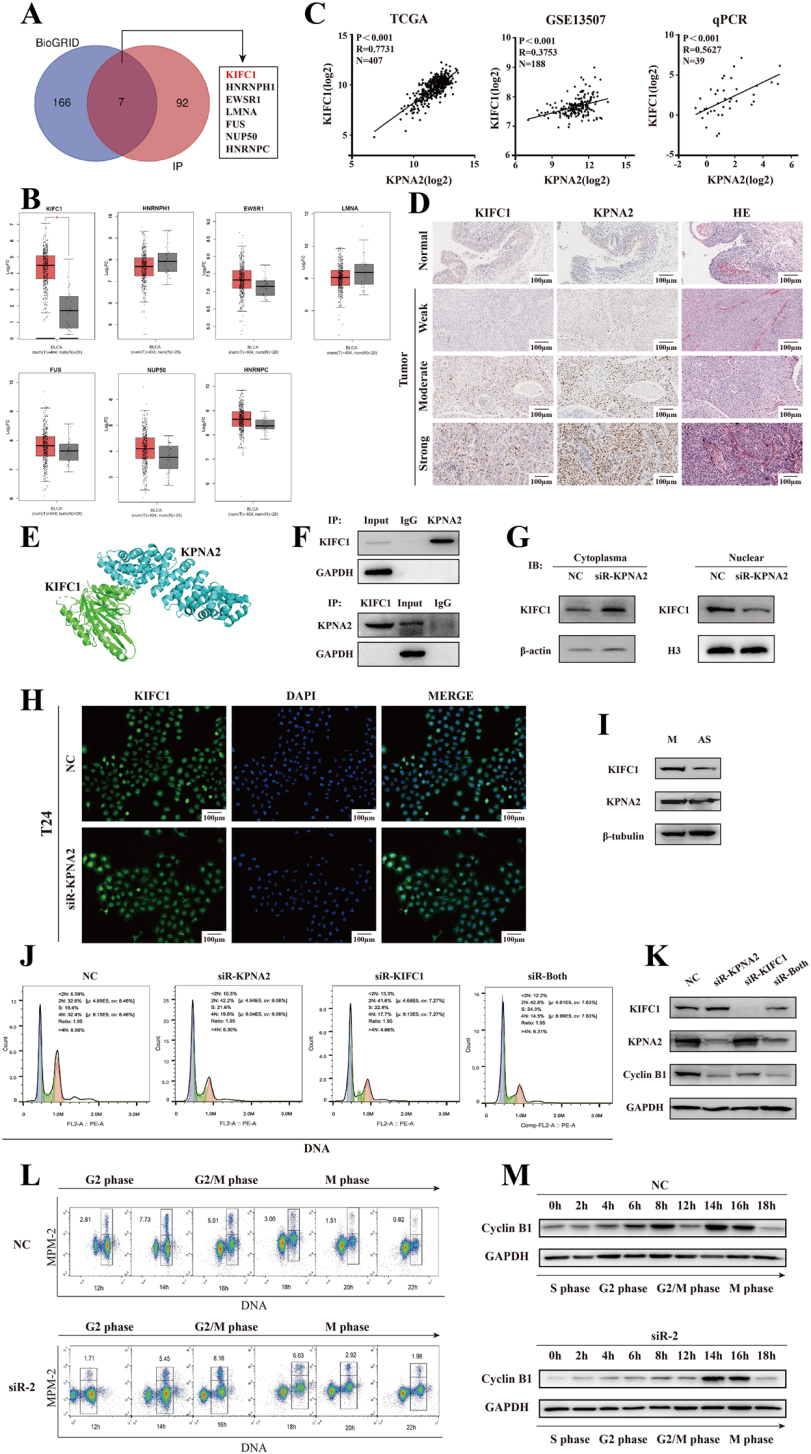
*KPNA2* imports KIFC1 into the nucleus of BCa cells. **A** To identify the protein cargos of *KPNA2* in BCa cells, 173 potential cargos from the BioGRID database and the top 99 potential cargos from mass spectrometry analysis data were integrated using Venn Diagrams. Seven common potential cargo proteins (KIFC1, HNRNPH1, EWSR1, LMNA, FUS, NUP50, and HNRNPC) were chosen for further investigation. **B** The TCGA visualization database GEPIA was utilized to analyze the mRNA levels of the seven genes in BCa tissues and normal bladder tissues. Among these genes, KIFC1 showed a significant up-regulation in BCa tissues compared with normal bladder tissues. **C** Pearson correlation analysis of TCGA data revealed a positive correlation between the expression of KIFC1 and *KPNA2* in 426 BCa tissues (*r* = 0.7731, *p* < 0.001). Similarly, analysis of GSE13507 data showed a positive correlation between KIFC1 expression and *KPNA2* in 188 BCa tissues (*r* = 0.3753, *p* < 0.001). In addition, correlation analysis of KIFC1 and *KPNA2* mRNA levels in our 39 BCa tissues demonstrated a positive correlation between the two genes (*r* = 0.5627, *p* < 0.001). **D** The immunohistochemistry staining results revealed that KIFC1 expression was elevated in BCa tissues exhibiting intense *KPNA2* immunostaining, whereas KIFC1 expression was reduced in BCa tissues with faint *KPNA2* immunostaining. **E** The HDOCK database suggests that *KPNA2* can bind to KIFC1. **F** The co-immunoprecipitation assay was performed for the interaction of KIFC1 and *KPNA2* in BCa cells. **G**, **H** After knockdown of *KPNA2* in BCa cells, changes in the cytoplasmic and nuclear fractions of KIFC1 were determined by Western blotting and immunofluorescence. **I** After synchronizing the cells, the protein levels of KIFC1 and *KPNA2* in the mitotic cell lysates and inhibited mitotic cell lysates were determined using western blotting. The findings indicated that both KIFC1 and *KPNA2* were overexpressed during the M phase of BCa cells. **J**, **K** Knockdown of KIFC1 and/or *KPNA2* in BCa cells led to a notable reduction in the proportion of G2/M phase cells. Western blotting analysis revealed that the knockdown of KIFC1 and/or *KPNA2* led to a decrease in the cell cycle protein B1. **L** Dot plots of PI (DNA) vs FITC (MPM-2) showing cells in G2 and M-phase specifically during the time of mitotic exit in 5637-NC and 5637 KIFC1-siRNA2 cells. **M** The western blot showed cyclin B1 protein levels in synchronized 5637-NC and 5637 KIFC1-siRNA2 cells following release from thymidine block at G1/S boundary

To explore the procarcinogenic role of *KPNA2* in BCa, we utilized high-throughput mass spectrometry to identify dysregulated *KPNA2* cargo proteins (Additional file 5: Table S4). Combining this data with the BioGRID database, we identified seven potential cargo proteins: KIFC1, HNRNPH1, EWSR1, LMNA, FUS, NUP50, and HNRNPC (Fig. 5A). KIFC1 levels were significantly elevated in 407 BCa samples, while other mRNAs were comparable to those in normal tissues (Fig. 5B). A positive correlation between KIFC1 and *KPNA2* expression in BCa tissues was confirmed by TCGA (*r* = 0.7731, *p* < 0.001) and GEO Profile GSE13507 databases (*r* = 0.3753, *p* < 0.001), further corroborated by RT-qPCR analysis of 39 BCa and adjacent normal tissues (*r* = 0.5627, *p* < 0.001) (Fig. 5C). Immunohistochemical staining demonstrated higher KIFC1 expression with strong *KPNA2* staining and lower expression with weak staining (Fig. 5D). These findings validate the correlation between KIFC1 and *KPNA2* in BCa. HDOCK analysis predicted strong binding between *KPNA2* and KIFC1 (Fig. 5E). Corroborated by co-immunoprecipitation (Fig. 5F). *KPNA2* knockdown in BCa cells increased cytoplasmic and decreased nuclear KIFC1 levels, but the total amount of KIFC1 does not change significantly (Additional file 1: Fig. S3), underscoring its role in nuclear transport (Fig. 5G-H). In asynchronous and nocodazole-arrested mitotic 5637 cells, both KIFC1 and *KPNA2* levels increased during mitosis (Fig. 5I). Additional experiments showed that BCa cell numbers in the G2/M phase decreased following *KPNA2* and/or KIFC1 knockdown, along with reduced cyclin B1 levels (Fig. 5J-K).

### Overexpression of KIFC1 and *KPNA2* is significantly associated with the pathology grade of patients with BCa

To evaluate the clinical significance of KIFC1 and *KPNA2*, we analyzed their correlations with clinical features in 370 BCa cases (Table 1). Patients were classified into low (*n* = 185) and high (*n* = 185) expression groups on the basis of median expression levels. Abnormal KIFC1 and *KPNA2* expression correlated significantly with pathology grade (*p* < 0.001) but not with age, sex, or 5-year survival (Table 1). Furthermore, we detected KIFC1 and *KPNA2* expression and localization through immunohistochemistry and analyzed their clinicopathological correlations (Table 2). The low KIFC1 expression group had smaller tumor sizes (*p* = 0.02), while the low *KPNA2* group was predominantly at the Tis-T1 stage (*p* = 0.005). No significant correlations with age, gender, pN status, or survival were observed, consistent with TCGA findings. These results establish a correlation between KIFC1 and *KPNA2* expression and aggressive tumorigenesis in BCa.

**Table 1:** Correlation of KIFC1 and *KPNA2* with clinicopathological characteristics of patients with BCa (TCGA)

Clinic-pathologic variables	No. of cases	KIFC1 expression		*χ*^2^	*Ρ*	*KPNA2* expression		*χ*^2^	*Ρ*^(1)^
		Low	High			Low	High		
All cases	370	185	185			185	185		
Sex									
Male	274	132	142	1.407	0.236	135	139	0.225	0.635
Female	96	53	43			50	46		
Age (years)									
≤ 60	96	49	47	0.056	0.813	54	42	2.026	0.155
> 60	274	136	138			131	143		
Pathology grade									
Low grade	20	17	3	10.360	0.001	19	1	17.13	0.000
High grade	350	168	182			166	184		
pT status									
Tis–T1	4	2	2	0.000	1.000	2	2	0.000	1.000
T2–T4	366	183	183			183	183		
pN status									
N−	247	125	122	0.229	0.632	125	122	0.110	0.741
N +	123	59	64			60	63		
pM status									
Mx	182	80	102	5.382	0.068	84	98	2.166	0.339
M0	180	100	80			97	83		
M1	8	5	3			4	4		
Years of survival									
< 5 years	325	164	161	0.228	0.633	159	166	1.240	0.266
≥ 5 years	45	21	24			26	19		

^(1)^(*p* < 0.05 was considered to be stoically significant)

**Table 2:** Correlation of KIFC1 and *KPNA2* with clinicopathological characteristics of patients with BCa (IHC)

Clinic-pathologic variables	No. of cases	KIFC1 expression		*χ*^2^	*Ρ*	*KPNA2* expression		*χ*^2^	*Ρ*^(1)^
		Low	High			Low	High		
All cases	52	13	39			12	40		
Sex				0.0	1.0			0.596	0.44
Male	44	11	33			11	33		
Female	8	2	6			1	7		
Age (years)				0.578	0.447			0.032	0.858
≤ 60	12	2	10			3	9		
> 60	40	11	29			9	31		
Tumor size(cm)				5.419	0.02			0.004	0.95
< 3	9	5	4			2	7		
≥ 3	43	8	35			10	33		
Primary tumor stage				3.258	0.071			7.823	0.005
Tis–T1	14	6	8			7	7		
T2–T4	38	7	31			5	33		
pN status				2.261	0.133			0.157	0.692
N−	46	13	33			11	35		
N +	6	0	6			1	5		
Pathology grade				0.693	0.405			0.849	0.357
Low grade	2	0	2			1	1		
High grade	50	13	37			11	39		
Years of survival				0.447	0.578			0.924	0.336
≤ 5 years		9	31			8	32		
> 5 years		4	8			4	8		

^(1)^(P < 0.05 was considered to be stoically significant)

### KIFC1 promotes cell growth of BCa cell in vitro and in vivo

To investigate *KIFC1*’s role in BCa, we assessed its expression in normal uroepithelial cells and six BCa cell lines using RT-qPCR and immunoblotting. KIFC1 was upregulated in all BCa lines, particularly in SW780 and 5637, while levels decreased in T24 and UMUC-3 (Additional file 1: Fig. S2A), consistent with findings from KH Xiao’s team [18]. We silenced KIFC1 in SW780 and 5637, and overexpressed it in UMUC-3 and T24, analyzing gene and protein levels (Additional file 1: Fig. S2B-C, Fig. S2E-F). Growth curve analyses showed that silencing KIFC1 reduced growth in 5637 and SW780 (Additional file 1: Fig. S2D, Fig. S2H-J), whereas overexpression enhanced proliferation in UMUC-3 and T24 (Additional file 1: Fig. S2G). In a xenograft model, KIFC1 overexpression in UMUC-3 significantly increased tumor volume and weight by day 22 compared with controls (Additional file 1: Fig. S2K-M), confirming its role in tumor growth in vivo.

Given this link, we selected 5637 cells for synchronized experiments using siRNA-2. The 5637-NC and 5637-siRNA-2 cells were synchronized with a 36-h thymidine block (2 mM) and collected at intervals for two-color cell cycle analysis via PI staining. The G2/M phase group (4N) showed double the PI intensity of the G1 phase group (2N). An anti-MPM-2 antibody labeled with Alexa488 distinguished the G2 and M phases (Fig. 5L). Cyclin B1 expression was assessed by western blot (Fig. 5M). Results showed that 5637-siRNA-2 cells entered the G2/M phase slower than 5637-NC cells.

### MiR-26b-5p inhibits BCa cell growth in vivo by targeting *KPNA2*

**Fig. 6:**
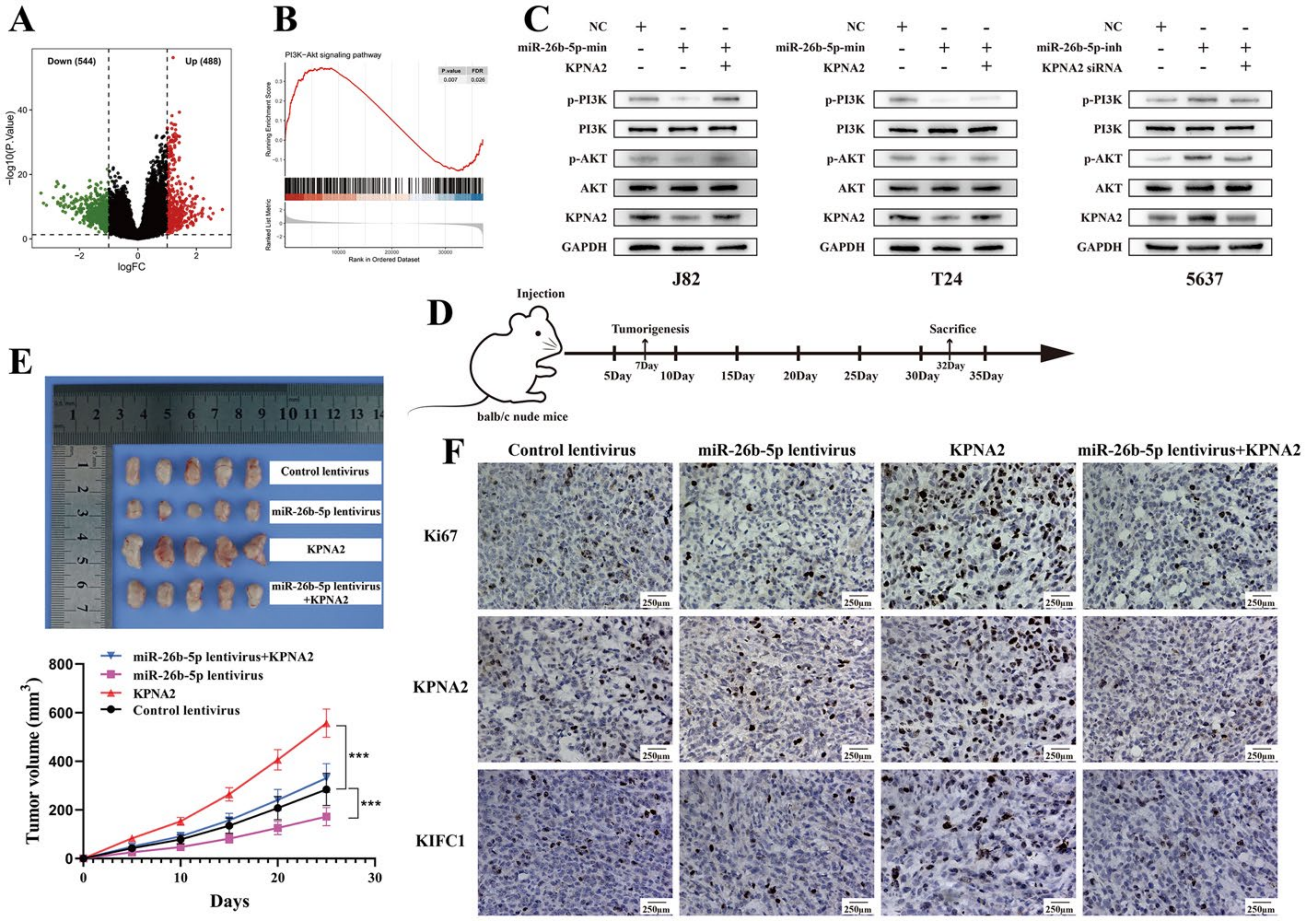
MiR-26b-5p inhibits BCa cell growth in *vivo* by targeting *KPNA2*. **A**, **B** On the basis of the median level of *KPNA2*, a distribution map of DEGs was generated, identifying a total of 1032 DEGs, with 544 upregulated and 488 downregulated. Gene Set Enrichment Analysis (GSEA) indicated that the DEGs are closely linked to the PI3K/AKT signaling pathway. **C** Western blotting to detect the expression of key genes of the PI3K/AKT pathway. **D** Nude Mouse Subcutaneous Tumor Model Diagram. **E** Measurement of tumor volume at specified time points and upon completion of the experiment, mice were euthanized, and the weight of subcutaneous tumors was recorded. **F** Immunohistochemical staining of Ki-67, KIFC1 and *KPNA2* in tumors implanted in mice. *, *p* < 0.05; **, *p* < 0.01; ***, *p* < 0.001

In our TCGA-BLCA analysis, we divided samples into high and low *KPNA2* expression groups, identifying 1032 differentially expressed genes (DEGs): 544 up-regulated and 488 down-regulated (Fig. 6A). Gene set enrichment analysis (GSEA) linked these DEGs to 46 pathways, notably the PI3K/AKT signaling pathway, essential for cell growth and survival [19, 20] (Fig. 6B). Western blot showed *KPNA2* knockdown reduced phosphorylated PI3K and AKT levels, while overexpression increased them, without affecting total protein levels (Fig. 6C). To examine the in vivo roles of miR-26b-5p and *KPNA2*, we developed a nude mouse xenograft model using cells cotransfected with control, miR-26b-5p, *KPNA2*, and both lentiviruses (Fig. 6D). Results showed that *KPNA2* overexpression diminished miR-26b-5p’s inhibitory effect on tumor growth, implying miR-26b-5p targets *KPNA2* to suppress tumors (Fig. 6E). IHC analysis of paraffin-embedded tumors revealed lower *KPNA2* and Ki-67 expression in the miR-26b-5p overexpression group and higher expression in the *KPNA2* overexpression group. (Fig. 6F). These findings, consistent with in vitro experiments, validate miR-26b-5p as a tumor suppressor in BCa by targeting *KPNA2*.

### *KPNA2* proteins encapsulated in exosomes have the potential to act as tumor markers and regulate fibroblast activation

**Fig. 7:**
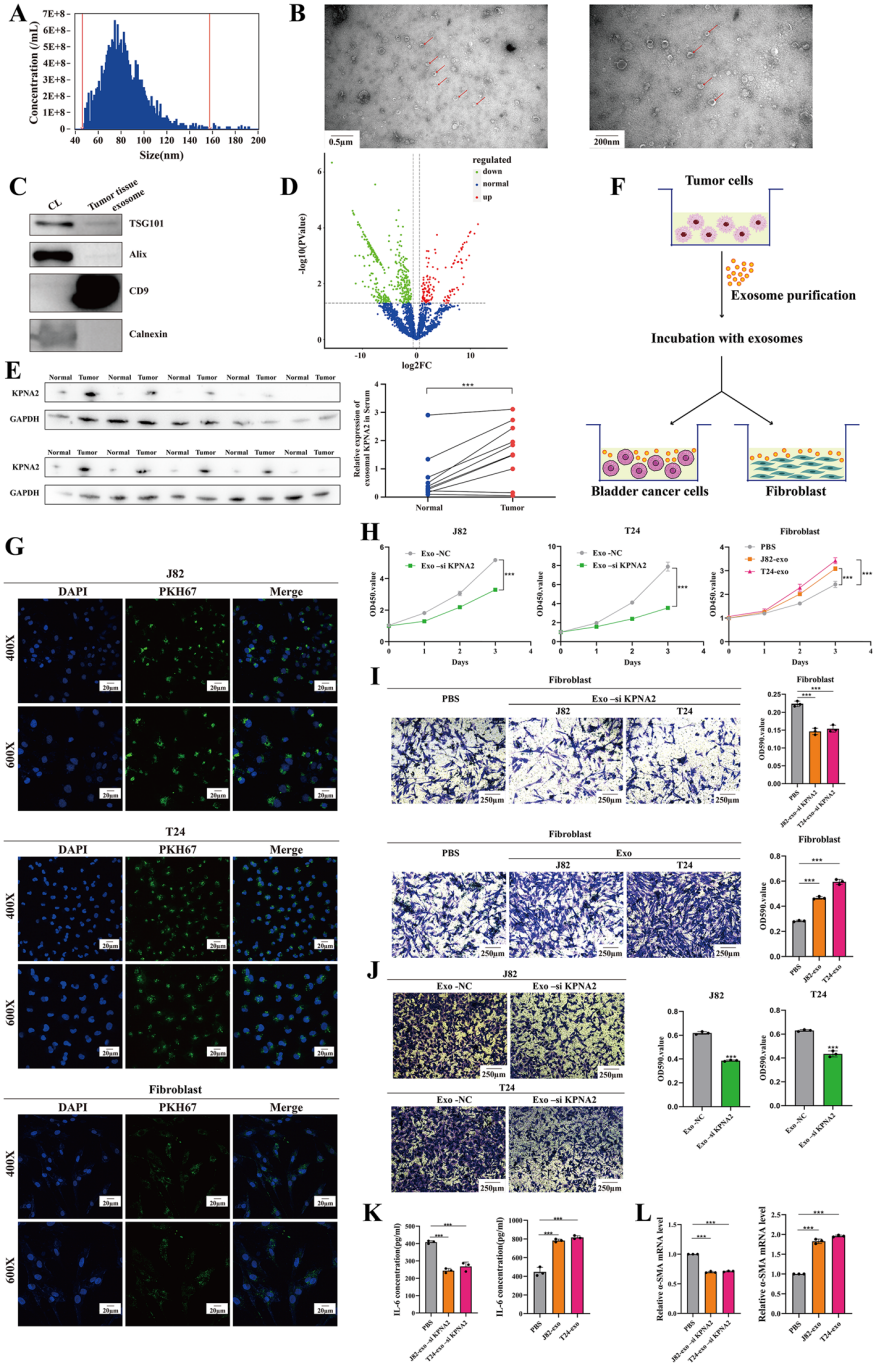
*KPNA2* proteins encapsulated in exosomes have the potential to act as tumor markers and regulate fibroblast activation. **A** Exosome particle size analysis. **B** Electron microscopic identification of exosomes. **C** Exosome marker western blot identification. **D** Tissue sequencing of exosomal proteins identifies *KPNA2* proteins encapsulated within exosomes. **E** Patients with tumors have significantly higher levels of *KPNA2* protein in serum exosomes compared with normal individuals. **F** Exosome coculture model diagram. **G** Representative microscope images of J82, T24 and fibroblast cytosolic uptake of PKH67-labelled exosomes are shown. **H** Analysis of relative proliferation rate in CCK-8 assay after coculture with different levels of *KPNA2*-containing exosomes. **I** Fibroblasts show enhanced migratory ability after coculturing with exosomes containing *KPNA2* compared with the PBS group or Exo-si*KPNA2* group. **J** In Transwell assays, the migration of J82 and T24 cells cocultured with exosomes containing different levels of *KPNA2* was examined. **K** Fibroblasts secrete more IL-6 after coculturing with exosomes containing *KPNA2* compared with the PBS group or Exo-si*KPNA2* group. **L** Fibroblasts showed higher levels of α-SMA expression after coculturing with exosomes containing *KPNA2* than the PBS or Exo-si*KPNA2* groups. *, *p* < 0.05; **, *p* < 0.01; ***, *p* < 0.001

Previous studies have highlighted the essential role of exosomes in tumor metastasis [21, 22]. We collected three pairs of BCa tissues and their matched normal tissues and assessed the expression of *KPNA2* in bladder exosomes using advanced tissue exosome protein proteomic sequencing technology (Fig. 7A-D). Several studies have demonstrated the utility of exosome-based liquid biopsy for identifying non-invasive biomarkers [23]. Our analysis revealed significantly elevated serum exo-*KPNA2* levels in tumor patients compared with healthy individuals (Fig. 7E). These findings suggest that exo-*KPNA2* may serve as a potential BCa biomarker, promising future applications in liquid biopsies.

Exosomal signaling molecules influence neighboring cells and the tumor microenvironment [24, 25]. To evaluate the effect of exo-*KPNA2* on adjacent cells, we isolated and labeled exosomes with PKH67 dye from the supernatants of T24 and J82 cells, then coincubated them with recipient cells (Fig. 7F). PKH67 labeling confirmed efficient exosome uptake by BCa cells (Fig. 7G). Cells treated with exo-NC exhibited increased proliferation and invasion compared with those treated with exo-si*KPNA2* (Fig. 7H, J). Cancer-associated fibroblasts promote metastasis by secreting cytokines, such as IL-6. Coculturing with *KPNA2*-rich exosomes significantly upregulated the α-SMA levels in fibroblasts compared with the PBS control group or the exo-si*KPNA2* treatment group, indicating the activation of CAFs (Fig. 7L). Similarly, these activated fibroblasts showed enhanced proliferation, migration, and elevated IL-6 levels compared with the PBS control group or the exo-si*KPNA2* group (Fig. 7H, I, K). Immunofluorescence detection of α-SMA showed that after coculturing fibroblasts with *KPNA2*-rich exosomes, α-SMA expression increased, and the cell morphology exhibited structural disorganization, consistent with the CAF phenotype characteristics (Additional file 1: Fig. S4). These findings suggest that exo-*KPNA2* from BCa cells can activate fibroblasts, potentially promoting tumor progression and metastasis.

## Discussion

Managing advanced BCa remains challenging due to limited effective therapies. Understanding BCa’s molecular mechanisms is essential for new therapeutic developments.

*KPNA2*, also known as Rch1 or hSRP1, plays a significant role in cell signaling and nuclear-cytoplasmic transport, affecting tumor progression. However, its precise functions are not fully understood. Our findings show *KPNA2* upregulation in BCa tissues, especially in exosomes. Mechanistically, *KPNA2* promotes BCa cell proliferation by interacting with KIFC1 and activating the PI3K-AKT pathway, a process inhibited by miR-26b-5p. Notably, exosomes with *KPNA2* transfer to fibroblasts, converting them into CAFs and creating a supportive environment for tumor progression [26, 27]. These results underscore exo-*KPNA2*’s role in BCa progression and its potential as a biomarker and therapeutic target.

MiR-26b-5p, a member of the miR-26b family, regulates cellular function by binding to target mRNA and modulating expression. Most studies have shown that reduced levels of miR-26b-5p in cancer affect genes associated with proliferation, invasion, metastasis, and apoptosis [28–34]. Our research revealed that in BCa tissues, miR-26b-5p expression is significantly diminished, making it a critical miRNA targeting *KPNA2*, and its overexpression inhibits BCa cell growth.

The primary drivers of tumor cell proliferation are accelerated growth and resistance to apoptosis. KIFC1 is a microtubule-associated protein essential for spindle formation and chromosome segregation during mitosis [35, 36]. Our study revealed that the *KPNA2*-interacting protein KIFC1 is upregulated in BCa, leading to tumorigenesis. Decreased expression of KIFC1 delays entry into the G2/M phase, while increased expression accelerates this entry. The G2/M transition is regulated by the cyclin B-Cdk1 complex; elevated cyclin B leads to premature M phase entry, disrupting division and promoting tumorigenesis, whereas decreased levels delay entry, slowing proliferation [37, 38]. These findings suggest that KIFC1 enhances BCa proliferation by hastening G2/M phase entry. Additionally, pathway enrichment analysis indicates that *KPNA2* may influence tumor progression through the PI3K-AKT pathway, consistent with Xiao et al.’s findings [18].

Notably, we found *KPNA2* in BCa tissues encapsulated within exosomes, protecting it from enzymatic degradation and potentially disrupting cellular communication. This mechanism may induce malignant traits in normal bladder epithelial cells, promoting tumor progression [3, 39]. Exosomes offer advantages in BCa biomarker studies over traditional serological markers by encapsulating diverse diagnostic components, such as oncogenic proteins and mutated DNA, which is valuable given tumor heterogeneity [40, 41]. Identifying *KPNA2* in BCa-derived exosomes positions it as a promising novel marker [9, 10].

Increasing evidence suggests that exosomes are crucial for mediating communication between cancer and stromal cells [42, 43]. Our study on the transfer of exosomal *KPNA2* to fibroblasts and their conversion into CAFs unveils a novel mechanism promoting BCa metastasis. CAFs enhance tumor progression by remodeling the extracellular matrix and secreting cytokines like IL-6, creating a supportive niche [26, 44]. *KPNA2* overexpression boosts α-SMA expression, a CAF marker, and enhances fibroblast proliferation and migration. However, the precise mechanism remains unclear and warrants further investigation to identify new BCa therapy targets.

In conclusion, our findings suggest that *KPNA2* promotes BCa progression by activating the PI3K/AKT pathway through interactions with KIFC1, a process inhibited by miR-26b-5p, exosomal *KPNA2* from BCa cells is a promising diagnostic marker and therapeutic target. However, the precise mechanisms through which *KPNA2* promotes metastasis, drug resistance, and fibroblast activation in BCa remain unclear and will be further investigated in our future study.

## Supplementary Information

Additional file 1.

Additional file 2.

Additional file 3.

Additional file 4.

Additional file 5.

Additional file 6.

## Data Availability

The original data of the study including gels and blots can be found in the article/ Additional file 6, and further inquiries can be directed to the corresponding authors.

## Abbreviations

BCa: Bladder cancer
KPNA2: Karyopherin a2
KIFC1: Kinesin Family Member C1
PI3K: Phosphoinositide 3-kinase
AKT: Protein Kinase B
CAFs: Cancer-associated fibroblasts
IL-6: Interleukin-6
miRNA: MicroRNA
mRNA: Messenger RNA
CT: Cycling threshold
IHC: Immunohistochemistry
CCK-8: Cell Counting Kit-8
PI: Propidium iodide
