# Supplementary material for: Tumor-derived exosomal KPNA2 activates fibroblasts and interacts with KIFC1 to promote bladder cancer progression, a process inhibited by miR-26b-5p
Source: Cell Mol Biol Lett. 2025 Feb 16;30:20. doi: 10.1186/s11658-025-00687-w (PMC11830183; doi:10.1186/s11658-025-00687-w)
Supplement: Supplementary file 1 — Additional file 1. [file 11658_2025_687_MOESM1_ESM.docx]

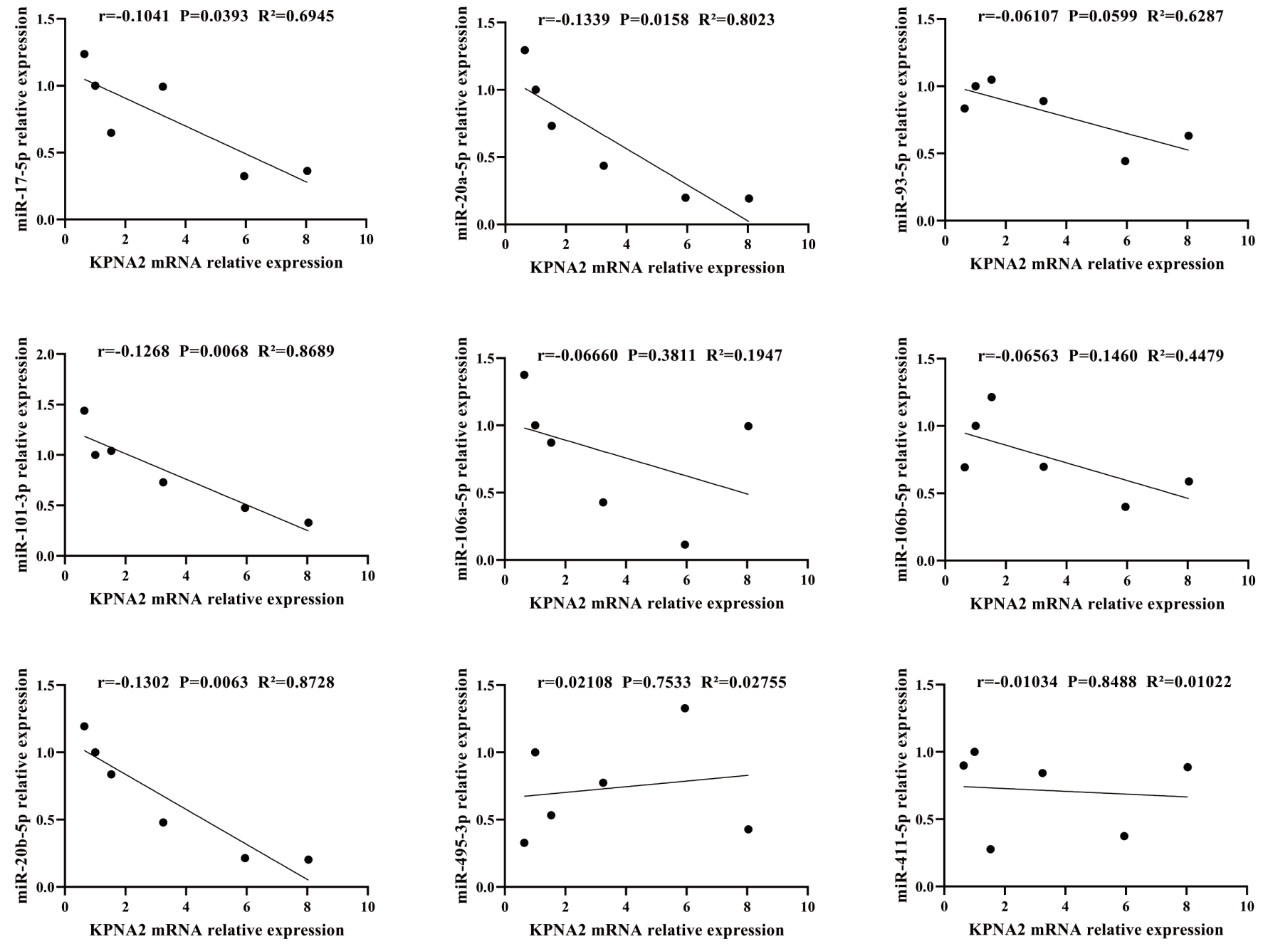


**Fig. S1 Screening of target miRNAs targeting regulation of KPNA2 in BCa cell lines.**


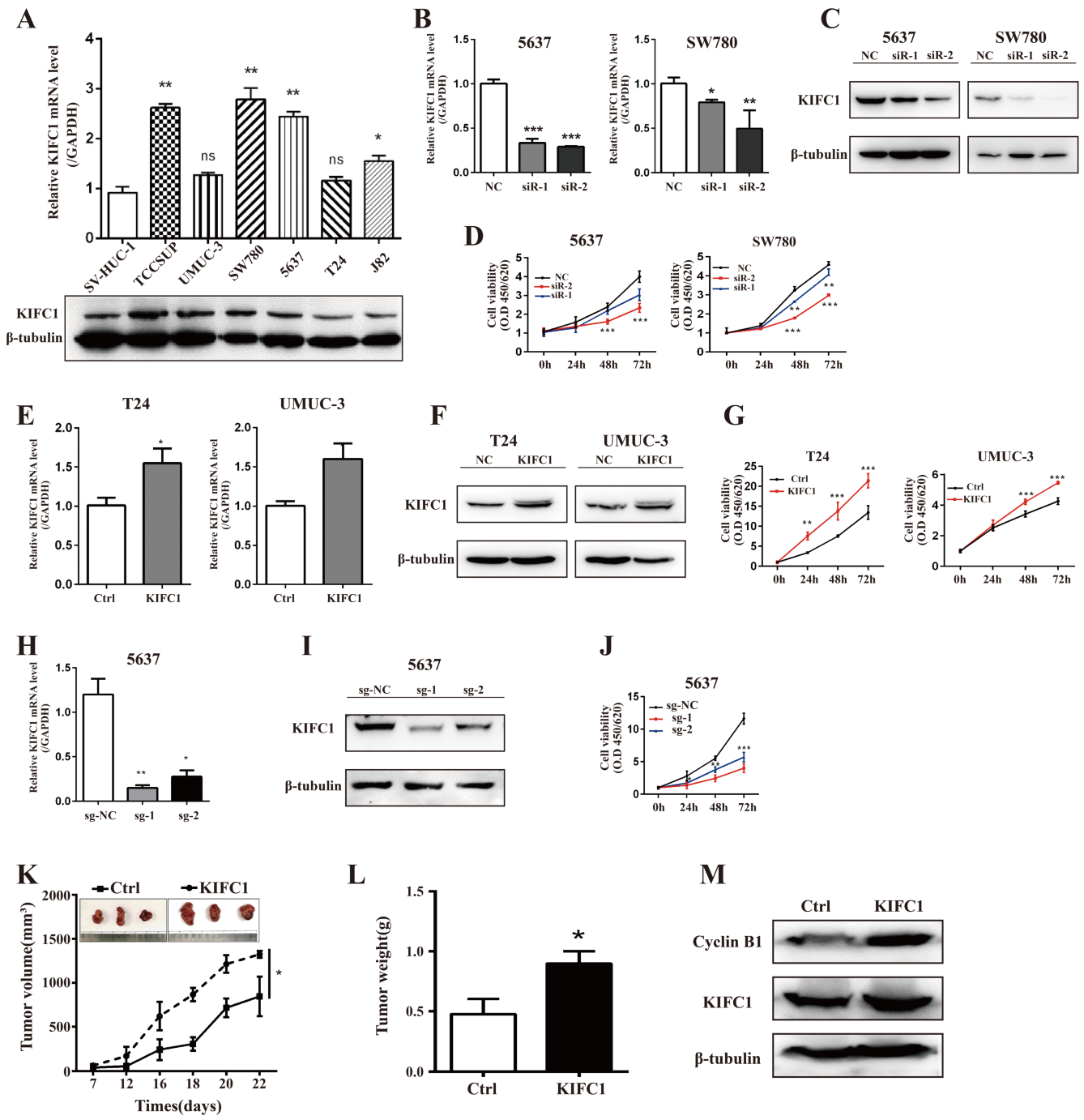


**Fig. S2 KIFC1 promotes cell growth of Bca cell in *vitro* and in *vivo*.**

**(A)** qPCR and Western blot results demonstrated significant upregulation of KIFC1 in TCCSUP, SW780, 5637, and J82 cell lines. **(B)** To further investigate, KIFC1-specific siRNAs (siR-1 and siR-2) were employed to transiently knock down KIFC1 in the 5637 and SW780 cell lines. The effectiveness of these siRNAs was confirmed by qPCR, showing a notable decrease in KIFC1 mRNA levels post-transfection. **(C)** Western blot analysis revealed a significant reduction in KIFC1 protein levels in the siRNA-treated groups compared to the negative control. **(D)** CCK-8 assays indicated that KIFC1 reduction suppressed cell proliferation. **(E)** RT-qPCR results showed upregulation of KIFC1 mRNA levels in T24 and UMUC-3 cells. **(F)** Following KIFC1 overexpression, Western blot analysis confirmed elevated KIFC1 protein levels in T24 and UMUC-3 cells. **(G)** CCK-8 results demonstrated that KIFC1 overexpression enhanced proliferation in T24 and UMUC-3 BCa cells. **(H)** Using CRISPR-Cas9 sgRNAs, KIFC1 was stably knocked down in the 5637 BCa cell line. qPCR confirmed the downregulation of KIFC1 after infection with KIFC1 sgRNAs. **(I)** Western blotting revealed decreased KIFC1 protein levels following infection with KIFC1 sgRNAs. **(J)** CCK-8 data indicated that KIFC1 knockdown inhibited cell growth. **(K)** The role of KIFC1 in *vivo* tumorigenesis was assessed using a subcutaneous tumor growth assay, where UMUC-3 cells with stable KIFC1 expression or negative control were injected into BALB/c nu/nu mice, and tumor volumes were measured at specified time points. **(L)** Upon completing the experiment, mice were euthanized, and the weight of subcutaneous tumors was recorded. **(M)** Western blot analysis indicated upregulation of KIFC1 and cyclin B1 in the KIFC1 stable expression tumor group compared to the control group, impacting the proliferation of BCa cells.


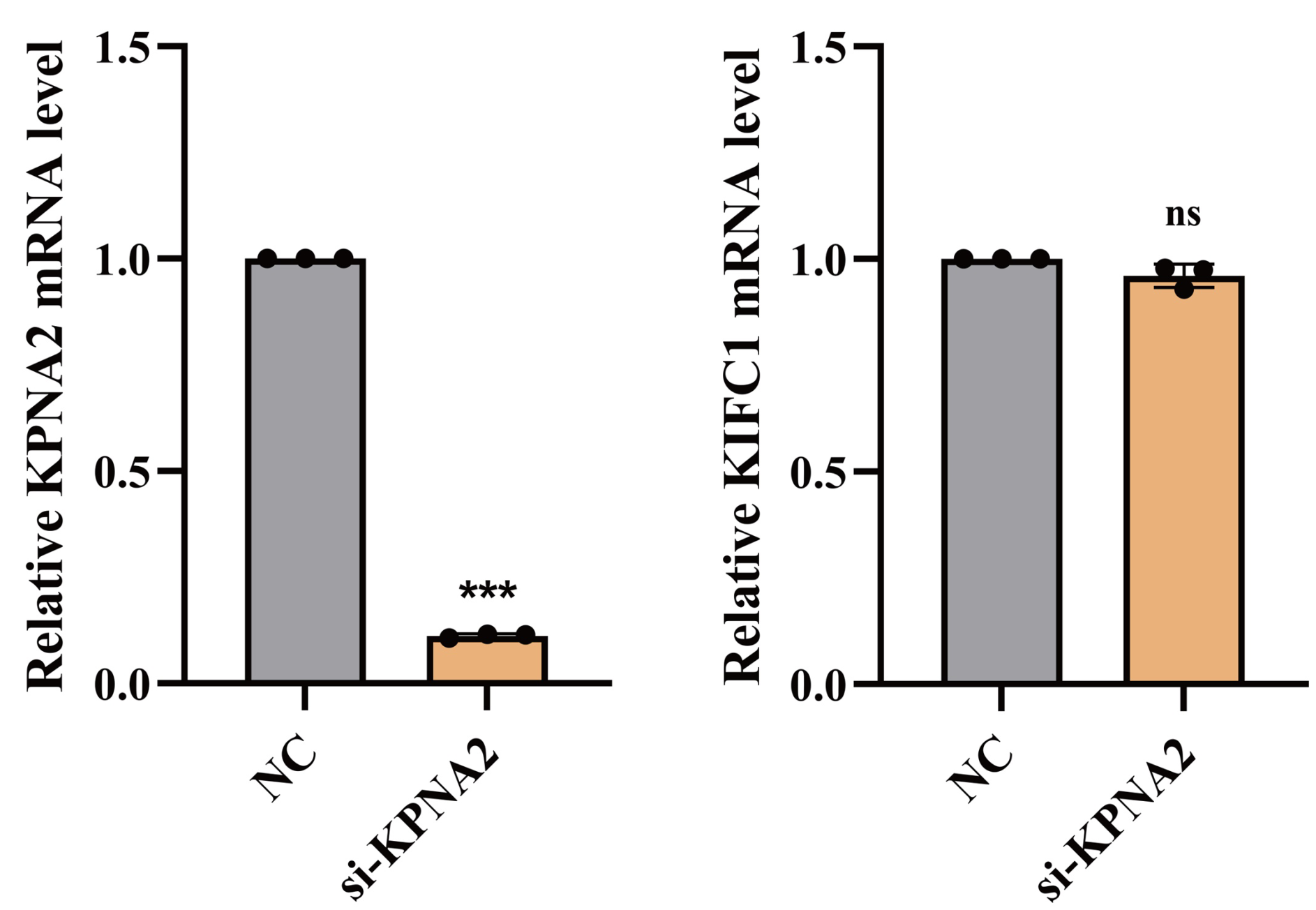


**Fig. S3 Knockout of KPNA2 did not significantly affect the total amount of KIFC1.**


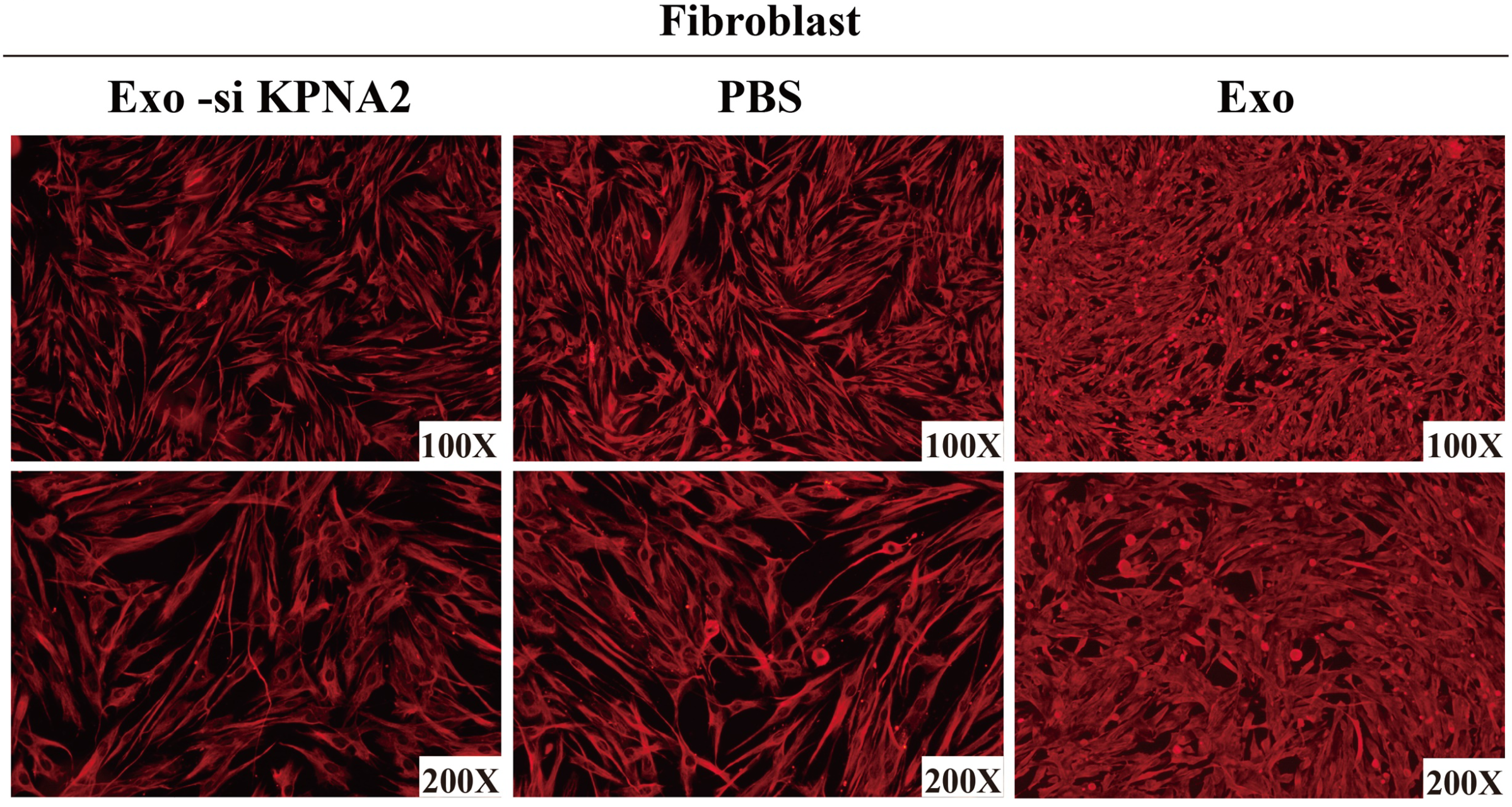


**Fig. S4 Immunofluorescence shows that exosomes rich in KPNA2 promote the transformation of fibroblasts into CAFs.**
