# Supplementary material for: Tumor-derived exosomal KPNA2 activates fibroblasts and interacts with KIFC1 to promote bladder cancer progression, a process inhibited by miR-26b-5p
Source: Cell Mol Biol Lett. 2025 Feb 16;30:20. doi: 10.1186/s11658-025-00687-w (PMC11830183; doi:10.1186/s11658-025-00687-w)
Supplement: Supplementary file 2 — Additional file 2. [file 11658_2025_687_MOESM2_ESM.docx]

Table S1. Primers used for RT-qPCR.

| **Target ID** | **Primer sequence 5’-3’** |
| --- | --- |
| **KIFC1-F** | GGTGCAACGACCAAAATTACC |
| **KIFC1-R** | GGGTCCTGTCTTCTTGGAAAC |
| **KPNA2-F** | CTGGGACATCAGAACAAACCAAG |
| **KPNA2-R** | ACACTGAGCCATCACCTGCAAT |
| **GAPDH-F** | CCACTCCTCCACCTTTGACG |
| **GAPDH-R** | CTGGTGGTCCAGGGGTCTTA |
| **miR-26b-5p** | GCGCAGTTCAAGTAATTCAG |
