# Supplementary material for: Tumor-derived exosomal KPNA2 activates fibroblasts and interacts with KIFC1 to promote bladder cancer progression, a process inhibited by miR-26b-5p
Source: Cell Mol Biol Lett. 2025 Feb 16;30:20. doi: 10.1186/s11658-025-00687-w (PMC11830183; doi:10.1186/s11658-025-00687-w)
Supplement: Supplementary file 3 — Additional file 3. [file 11658_2025_687_MOESM3_ESM.docx]

Table S2. Sequences of siRNAs for KIFC1 and KPNA2.

| **Target ID** | **Sequences** |
| --- | --- |
| **siR-KIFC1-1** | UAACUGACCCUUUAAGUCCUU |
|  | GGACUUAAAGGGUCAGUUAUU |
| **siR-KIFC1-2** | UGGUCCAACGUUUGAGUCCUU |
|  | GGACUCAAACGUUGGACCAUU |
| **siR-KPNA2** | GACUCAGGUUGUGAUUGAUTT |
|  | AUC AAUCACAACCUGAGUCTT |
| **hsa-miR-26b-5p inhibitor** | ACCUAUCCUGAAUUACUUGAA |
| **hsa-miR-26b-5p mimics** | UUCAAGUAAUUCAGGAUAGGU  CUAUCCUGAAUUACUUGAAUU |

Transcripts used for the KPNA2 plasmid: NM_001320611.2
