# Supplementary material for: Tumor-derived exosomal KPNA2 activates fibroblasts and interacts with KIFC1 to promote bladder cancer progression, a process inhibited by miR-26b-5p
Source: Cell Mol Biol Lett. 2025 Feb 16;30:20. doi: 10.1186/s11658-025-00687-w (PMC11830183; doi:10.1186/s11658-025-00687-w)
Supplement: Supplementary file 6 — Additional file 6. [file 11658_2025_687_MOESM6_ESM.docx]

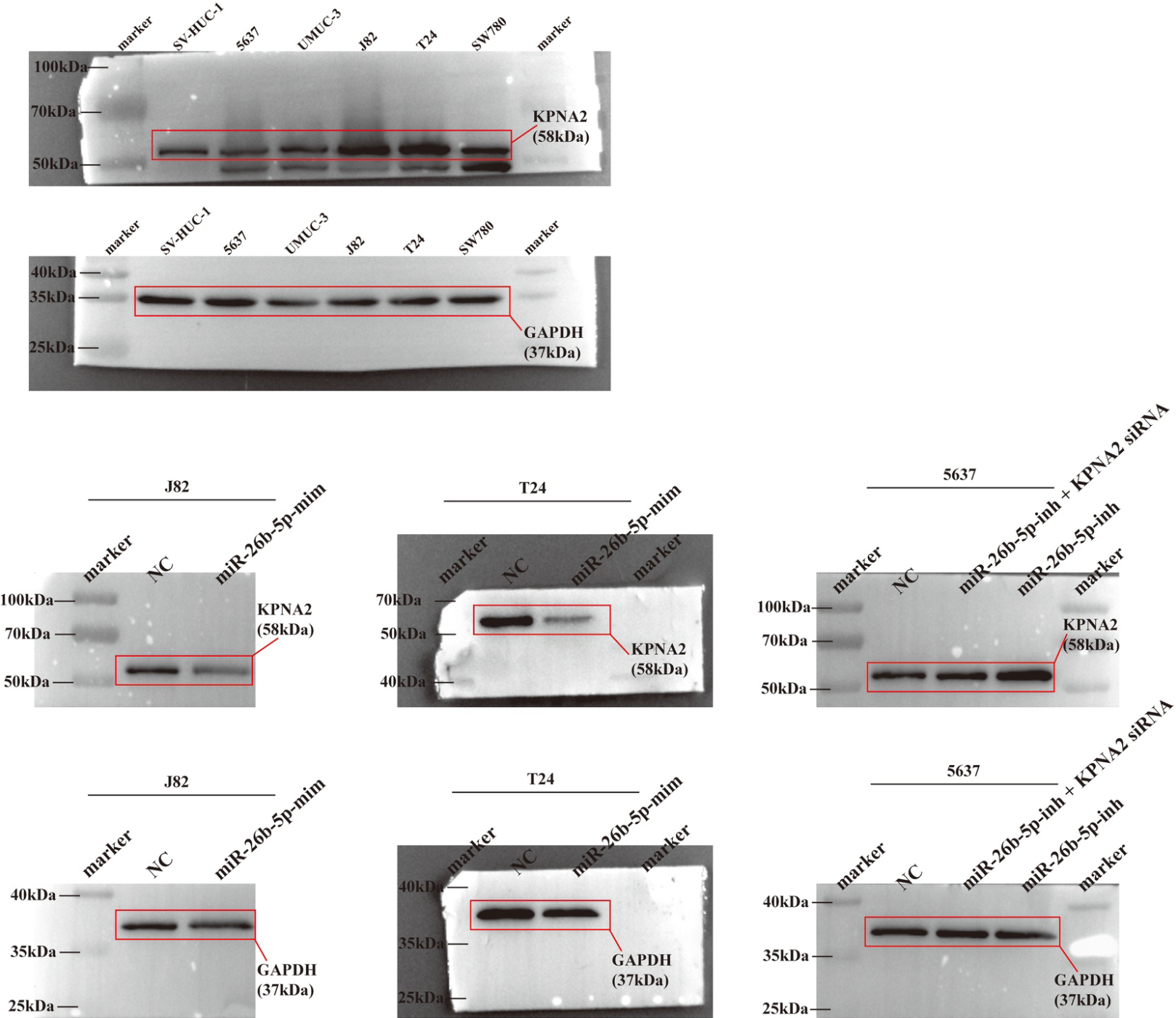


**Western blot merge figure (Related to Fig. 2)**


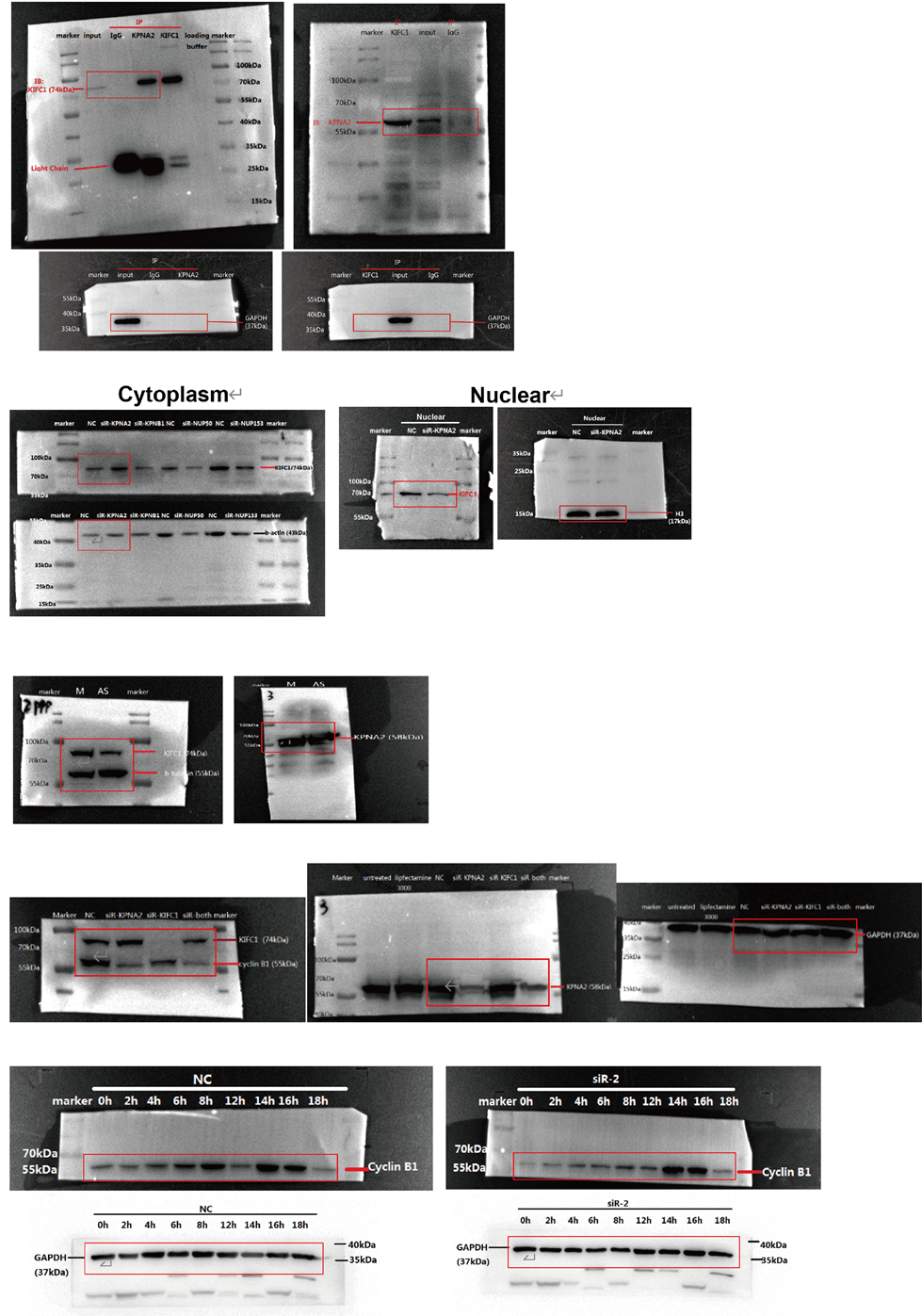
**Western blot merge figure (Related to Fig. 5)**


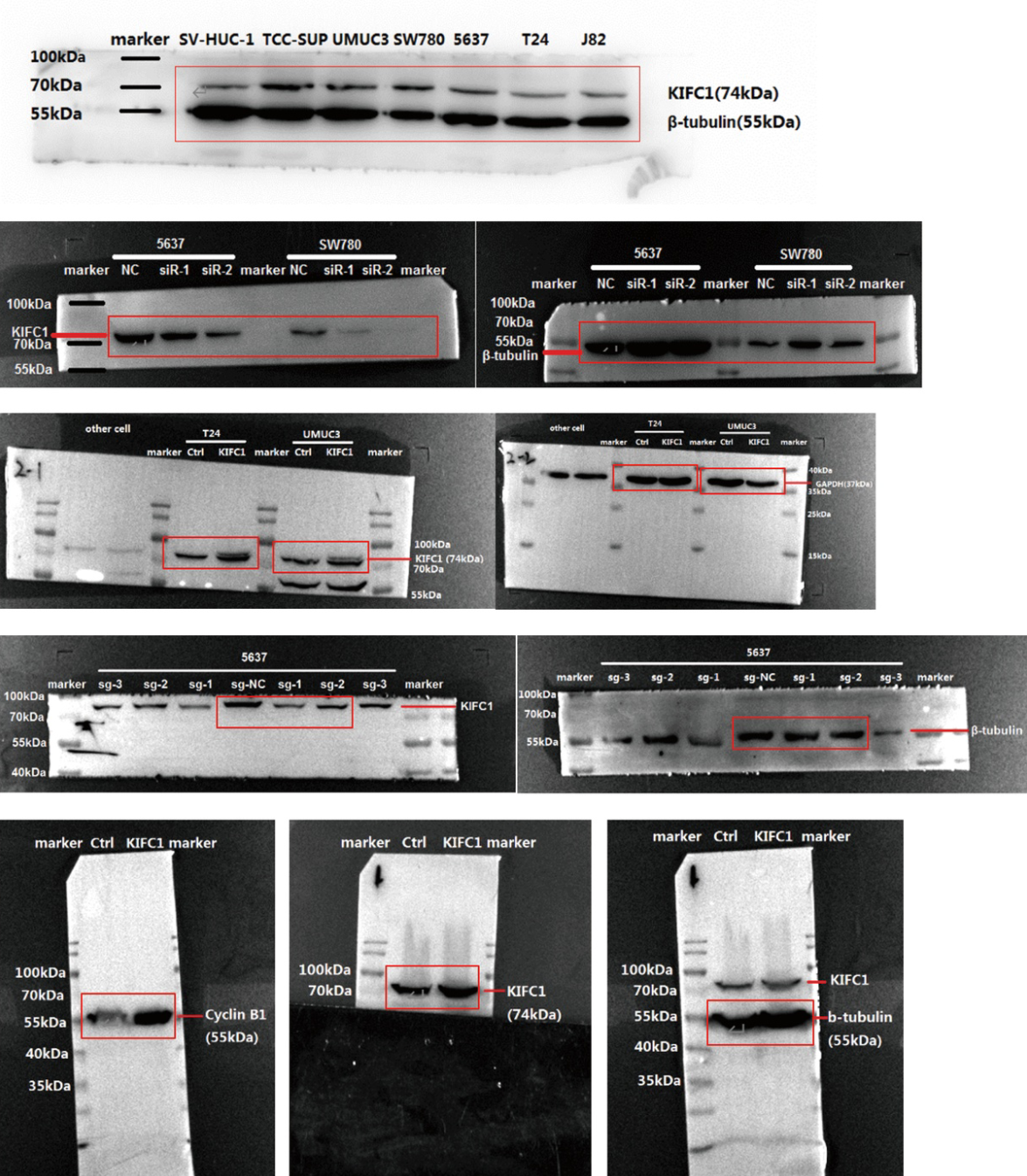


**Western blot merge figure (Related to Fig. S2)**


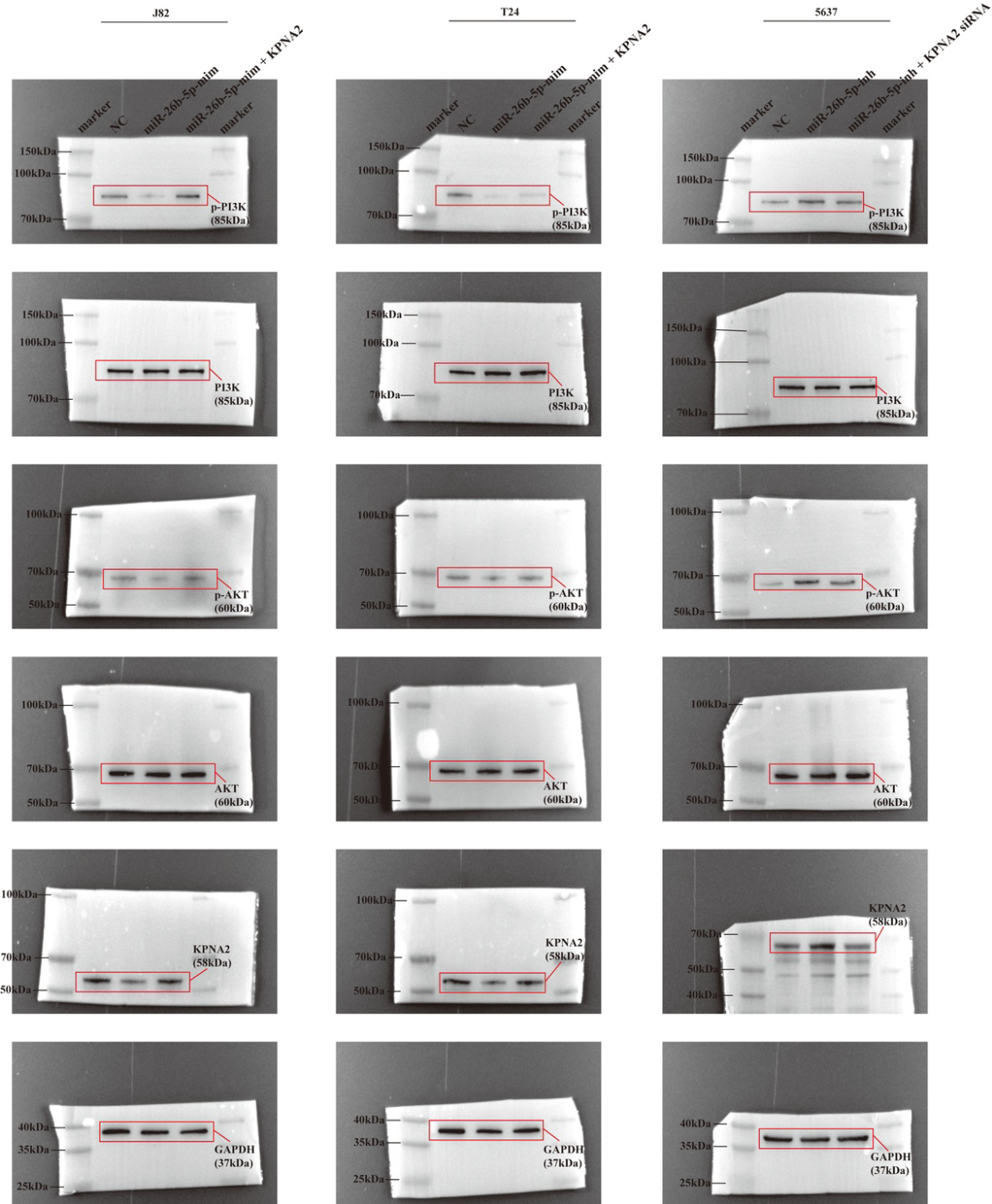


**Western blot merge figure (Related to Fig. 6)**


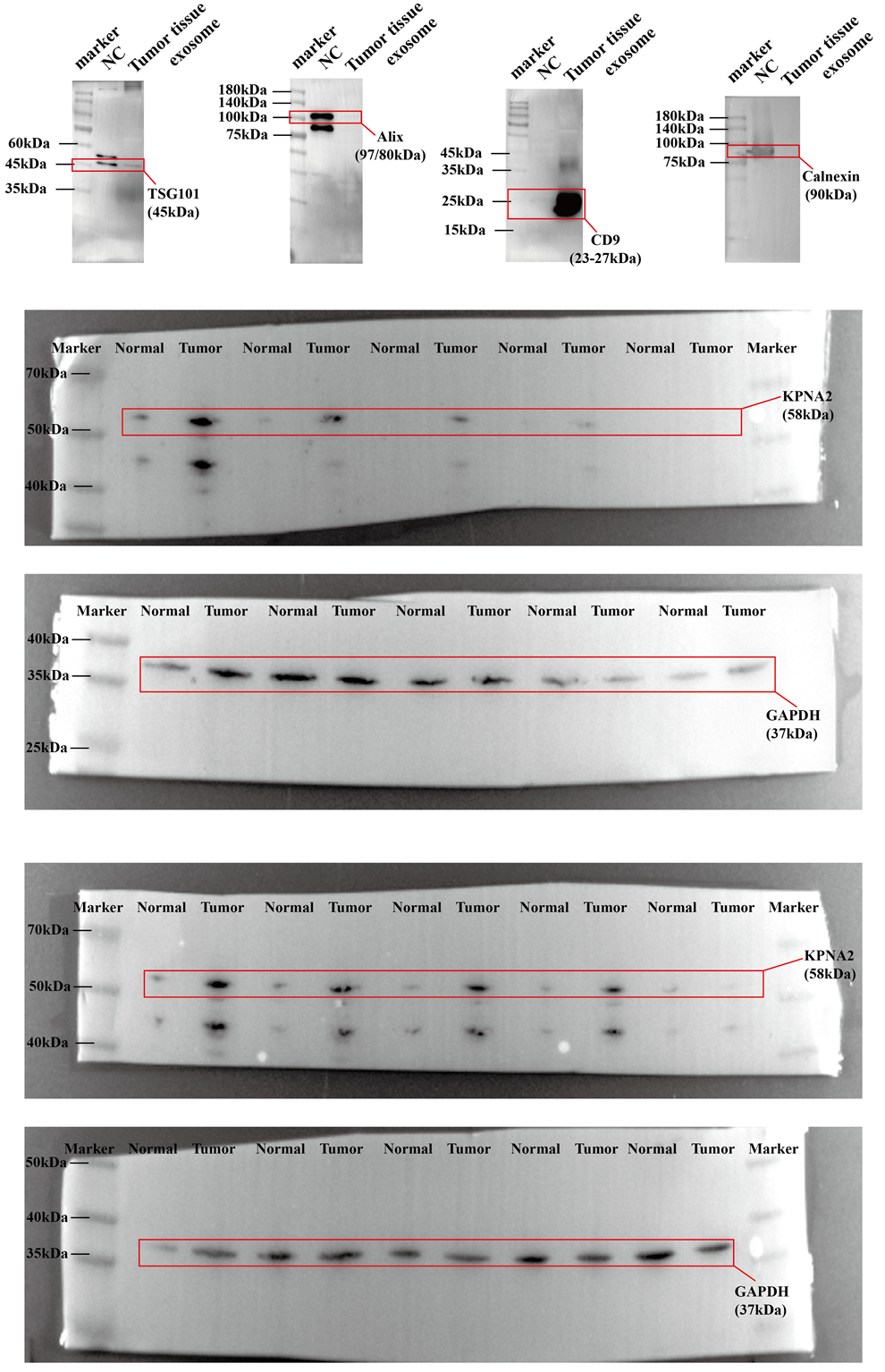


**Western blot merge figure (Related to Fig. 7)**
